# Association between delivery mode and postpartum psychiatric conditions: A national cohort study

**DOI:** 10.1097/AOG.0000000000006321

**Published:** 2026-05-21

**Authors:** Samantha L. Kruger, Nicola Perlman, Elizabeth B. Sherwin, Cecilia B. Leggett, Yasser El-Sayed, Deirdre J. Lyell, Elliott K. Main, Katherine L. Wisner, Pervez Sultan, Stephanie A. Leonard, Danielle M. Panelli

**Affiliations:** a Department of Obstetrics & Gynecology, Stanford University, Stanford, CA, USA; b Department of Psychiatry and Behavioral Sciences and Pediatrics, Children’s National Hospital, Washington, District of Columbia, USA; c Department of Obstetrics, George Washington University School of Medicine, Washington, District of Columbia, USA; d Division of Obstetric Anesthesiology, Department of Anesthesiology, Perioperative and Pain Medicine, Stanford University, Stanford, CA, USA

## Abstract

**OBJECTIVE:**

To evaluate the association between mode of delivery and incidence of new postpartum maternal psychiatric conditions in a national obstetric cohort.

**METHODS:**

This was an observational cohort study of individuals with singleton live births in the United States (2008–2022) identified from the Merative^™^ MarketScan^®^ Commercial Database using International Classification of Diseases, Ninth or Tenth Revision, Clinical Modification codes. Individuals with a psychiatric diagnosis preceding delivery and those with preterm births before 37 weeks of gestation were excluded. The exposure was mode of delivery, categorized as 1) spontaneous vaginal, 2) successful operative vaginal delivery (OVD), 3) planned cesarean, 4) unplanned cesarean without OVD attempt, 5) and unplanned cesarean after failed OVD attempt. The primary outcome was a new diagnosis of depression, anxiety, posttraumatic stress, or other serious psychiatric condition or a new antidepressant prescription within 6 months postpartum. Multivariable Poisson regression models with robust SEs estimated associations between mode of delivery and postpartum psychiatric conditions, first for any psychiatric condition and then separately by each condition.

**RESULTS:**

Of 934,524 included births, 66% were spontaneous vaginal, 4% were successful OVD, 14% were planned cesareans, 15% were unplanned cesareans without OVD attempt, and 1% were unplanned cesareans after failed OVD attempt. Compared with spontaneous vaginal births, postpartum psychiatric conditions were higher after planned cesarean births (11.4% vs 9.2%, adjusted risk ratio [RR] 1.19, 95% CI, 1.17–1.21), unplanned cesarean births without OVD (10.8% vs 9.2%, adjusted RR 1.16, 95% CI, 1.14–1.18), and unplanned cesarean births with failed OVD (10.9% vs 9.2%, adjusted RR 1.26, 95% CI, 1.18–1.35). Postpartum psychiatric condition risks were similar between individuals with spontaneous vaginal births and those with successful OVDs (9.2% vs 9.2%, adjusted RR 1.00, 95% CI, 0.97–1.03).

**CONCLUSION:**

Postpartum psychiatric conditions were increased after cesarean deliveries, planned or unplanned, but not after successful OVDs. Results suggest that cesarean delivery may be associated with an increased risk of psychiatric conditions and warrants closer surveillance postpartum.

Maternal psychiatric conditions are among the most common complications after childbirth.^1^ Approximately 85% of women experience some mood disturbance postpartum,^2,3^ and one in five will have more enduring symptoms that may result in a diagnosis of depression, anxiety, posttraumatic stress, or other serious mental health conditions such as bipolar disorder.^4,5^ Although many risk factors for postpartum psychiatric conditions have been identified, evidence on the role of mode of delivery is contradictory and limited.^2,3^

Prior studies have found that unplanned delivery interventions can result in patient traumatization, particularly with unplanned cesarean deliveries.^6,7^ However, evidence is lacking on the psychiatric sequelae of operative vaginal delivery (OVD) attempts. Vacuum- and forceps-assisted deliveries are an important component of labor management because they can expedite delivery for maternal or fetal indications while avoiding a second-stage cesarean delivery and its associated morbidities.^8^ Operative vaginal deliveries account for 3.4% (2.4–2.6% vacuum-assisted deliveries, 0.4–0.5% forceps-assisted deliveries) of all deliveries in the United States (2000–2018).^8–10^ An OVD attempt may be traumatic for birthing individuals, particularly when the goal is to expedite delivery in an emergency situation and the OVD attempt is unsuccessful. Prior studies have come to discordant conclusions on mental health outcomes with OVD.^2,11,12^

Robust, contemporary data evaluating the mental health consequences of all modes of delivery (including successful and unsuccessful OVD attempts) are needed. Therefore, we aimed to evaluate the association between mode of delivery and incident postpartum psychiatric diagnoses. We hypothesized that cesarean deliveries with and without OVD attempts, but not successful OVDs, would be associated with increased rates of postpartum psychiatric conditions compared with spontaneous vaginal birth.

## METHODS

This was an observational cohort study of individuals with singleton live births between 2008 and 2022 in the United States from the Merative^™^ MarketScan^®^ Commercial Database.^13^ The database includes reimbursed claims from inpatient and outpatient encounters, as well as outpatient pharmaceutical fills, covering more than 270 million individuals who are privately insured through employer-sponsored health insurance. Each record includes maternal age and region, diagnoses, procedures, and outpatient pharmaceutical prescription fills. We applied established methods to identify pregnancies, link maternal and neonatal records, and estimate gestational age.^14,15^ Eligibility was defined as individuals who were continuously enrolled in a health insurance plan from the estimated date of last menstrual period to 6 months (183 days) postpartum, including pharmaceutical coverage, with at least one health care encounter between 7 days and 6 months postpartum. The 6-month postpartum timepoint was selected on the basis of the rationale that the onset of trauma- or depression-related symptoms more than 6 months postpartum would be less likely to be directly attributable to the birth experience. In addition, extending follow-up beyond this period would increase the risk of selection bias attributable to loss of follow-up.

After identifying all linked live-birth records, we excluded individuals with preterm births (gestational age less than 37 weeks), multiple gestations, and nonstandard forceps deliveries. Those with deliveries before 37 weeks of gestation were excluded to minimize bias from gestational age restrictions on OVD attempts. Nonstandard forceps deliveries that were excluded included forceps used for after-coming head, mid or high forceps, rotational forceps, and combined vacuum and forceps, because these represent atypical clinical scenarios that are not comparable or generalizable with standard, contemporary obstetric practice. Their inclusion would also raise concern for information bias, because it questions the validity of the codes. To focus specifically on new-onset postpartum psychiatric conditions, individuals with prior psychiatric diagnoses or those who filled prescriptions for antidepressants (tricyclics, selective serotonin reuptake inhibitors, or neurosteroid antidepressants) between the last menstrual period and delivery were also excluded. This approach aimed to isolate the relationship between mode of delivery and psychiatric conditions by minimizing confounding by preexisting psychiatric conditions.

The exposure was mode of delivery, modeled as one variable in five mutually exclusive categories. This variable was categorized as spontaneous vaginal delivery (SVD), successful OVD with vacuum or forceps, planned cesarean delivery, unplanned cesarean delivery without OVD attempt, or cesarean delivery after failed OVD. Mode of delivery was determined using International Classification of Diseases, Ninth or Tenth Revision, Clinical Modification (ICD-9-CM and ICD-10-CM) diagnosis and procedure codes with birth encounters. The distinction between successful and failed OVD with these codes has been previously described.^10^ Spontaneous vaginal delivery was identified by no cesarean delivery, vacuum, or forceps codes linked to the birth encounter.^16^ Successful OVD was identified by a code for forceps or vacuum attempt and no cesarean delivery code. Planned cesarean delivery was identified by coding for cesarean delivery with coding for a contraindication to vaginal delivery, including placenta previa, vasa previa, and fetal malpresentation; if these codes were present without a code for cesarean delivery, those births were excluded from the analysis. Unplanned cesarean delivery was identified by coding representing an intrapartum course or trial of labor, indicating an attempt at vaginal delivery but with a cesarean delivery procedure code. Cesarean delivery after failed OVD was identified if a code for vacuum, forceps, or other instrumental delivery was present in addition to coding for a cesarean delivery procedure. Deliveries were classified as planned cesareans if they had both a cesarean delivery code and a code indicating a contraindication to vaginal delivery (eg, placenta previa, vasa previa, or fetal malpresentation). Cases in which these contraindication codes were present without a corresponding cesarean delivery code or in conjunction with a code indicating an OVD attempt were excluded. In addition, deliveries with a cesarean code but no labor-related codes or OVD attempt were classified as planned cesarean deliveries (Appendix 1, available online at http://links.lww.com/AOG/E671).

The primary outcome was postpartum psychiatric conditions diagnosed between day of delivery and 6 months postpartum. The conditions were depression, anxiety, posttraumatic stress (acute stress reaction, posttraumatic stress disorder [PTSD], adjustment disorder, other reactions to severe stress), and other serious psychiatric conditions (bipolar spectrum disorders, schizophrenia spectrum disorders, psychotic disorders, delusional disorders, and personality disorders), identified with ICD codes from inpatient and outpatient encounters (Appendix 1, http://links.lww.com/AOG/E671).^17,18^ We also defined depression to include a filled prescription for an antidepressant (tricyclic antidepressants, selective serotonin reuptake inhibitors, and neurosteroid antidepressants) in the absence of a corresponding ICD code suggesting alternative psychiatric condition (Appendix 2, available online at http://links.lww.com/AOG/E671).^19,20^ We evaluated the broad category of posttraumatic stress (rather than PTSD specifically) to comprehensively assess all potentially severe, traumatic responses to childbirth regardless of time of onset (eg, PTSD requires symptoms present for more than 1 month). Individuals with multiple psychiatric conditions were classified into all applicable psychiatric outcome groups; conditions were not mutually exclusive.

Baseline characteristics of individuals were compared by mode of delivery with the Pearson χ^2^ test for categorical variables and Kruskal–Wallis rank-sum test for continuous variables. Multivariable Poisson regression models with robust SEs estimated the associations between mode of delivery and any postpartum psychiatric condition with adjustment for confounders. Potential confounders were selected from causal diagrams informed by prior literature and included age, region, delivery year, class III obesity (body mass index [BMI, calculated as weight in kilograms divided by height in meters squared] 40 or higher) before delivery, substance use during pregnancy, gestational age at delivery, chronic hypertension, pregestational diabetes mellitus, and severe unexpected newborn complications.^2,21,22^ We adjusted for delivery year to account for temporal changes, including the potential contribution of the coronavirus disease 2019 (COVID-19) pandemic to mental health outcomes. Substance use was identified by codes for alcohol and drug use during pregnancy (tobacco, hallucinogens, cocaine, amphetamines, inhalants, or other unspecified drug use) (Appendix 3, available online at http://links.lww.com/AOG/E671). Although other potential confounding factors may affect postpartum mental health, we were intentional in selecting confounders that theoretically could affect every category within the mode of delivery exposure (eg, individuals with planned cesarean deliveries would not typically experience complications related to labor, such as intra-amniotic infection).

We ran a model evaluating any psychiatric diagnosis as an outcome and then repeated the same models for each individual diagnosis separately (depression, anxiety, posttraumatic stress, and serious mental illness). The adjusted risk ratios (RRs) with 95% CIs are reported. We secondarily assessed birth outcomes, including gestational age, induction of labor, postpartum hemorrhage, intraamniotic infection, severe maternal morbidity, and severe unexpected newborn complications, defined with established indices.^21^

Two sensitivity analyses were conducted: The first replicated the models with generalized estimating equations to account for potential clustering among births to one person; the second restricted the post-traumatic stress codes to ICD-10-CM years only (2016–2022) because of variability in coding practices across the ICD-9-CM to ICD-10-CM transition. A post hoc analysis aimed to address gaps in understanding the timing of postpartum psychiatric conditions. For this analysis, the timing of onset of each psychiatric diagnosis relative to childbirth was calculated according to when the initial ICD code or prescription occurred (0–90 or 91–180 days postpartum).

Statistical significance was set to *P*<.05. All analyses were conducted with R 4.4.1. The Stanford University IRB approved the study (IRB protocol 54362).

## RESULTS

**Fig. 1.**
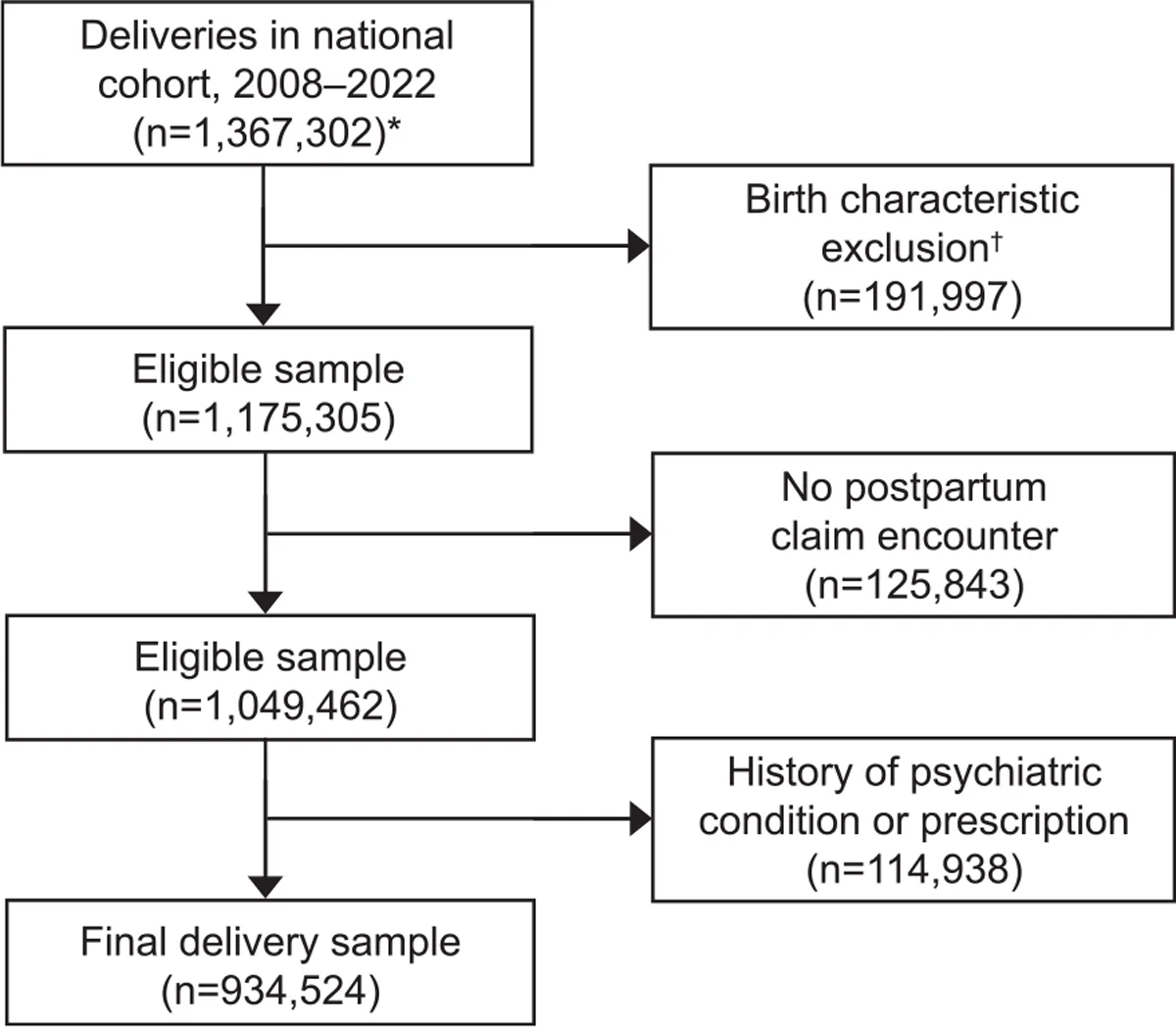
Study selection flow diagram. *Live births to individuals with continuous insurance from start of pregnancy through 6 months postpartum, defined as the date of the last menstrual period (LMP) through 183 days after the date of delivery (DOD); continuous prescription drug coverage from LMP through DOD plus 183 days; and continuous enrollment for the newborn from DOD to DOD plus 28 days. ^†^Preterm birth, multifetal gestation, mid or high forceps, rotational forceps, breech extraction forceps, forceps to after-coming head, combined operative vaginal delivery, or contraindication to cesarean delivery (malpresentation, vasa previa, or placenta previa) with no corresponding cesarean delivery code or contraindication to cesarean delivery (malpresentation, vasa previa, or placenta previa) in conjunction with a code indicating an operative vaginal delivery attempt.

Of 1,367,302 total births during the study period, 934,524 met inclusion criteria (Fig. 1). Of these, 66% were SVDs, 4% were successful OVDs, 14% were planned cesarean deliveries, 15% were unplanned cesarean deliveries with no OVD attempt, and 1% were cesarean deliveries after a failed OVD attempt. Characteristics varied by mode of delivery, with a higher proportion of individuals who underwent unplanned cesarean deliveries with and without OVD attempts having medical comorbidities such as class III obesity, pregestational diabetes, preeclampsia with severe features, and severe maternal morbidity (Table 1). A greater proportion of individuals undergoing planned cesarean deliveries were of advanced maternal age (35 years or older). The mean gestational age at delivery was 39 weeks for all exposure groups.

**Fig. 2.**
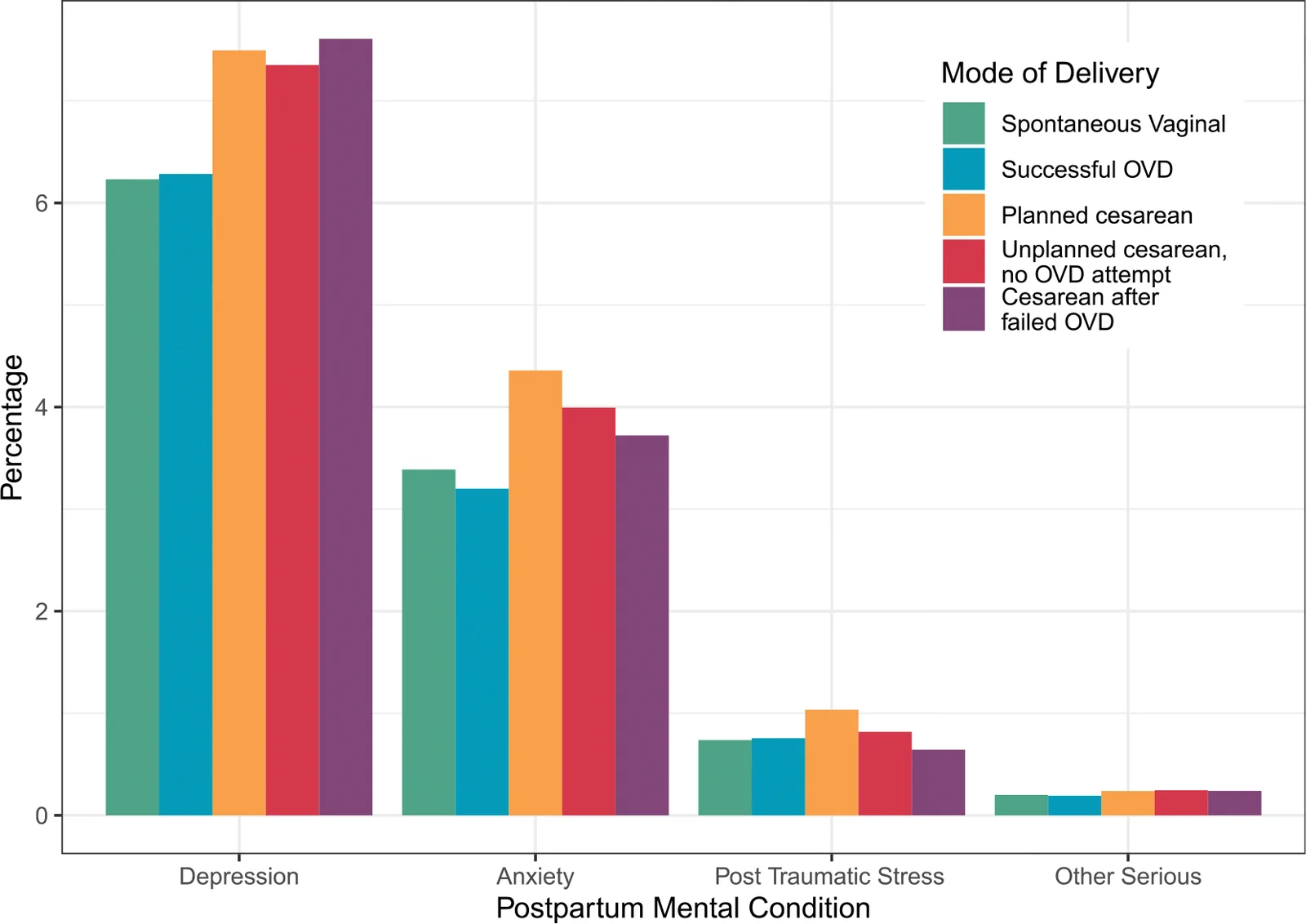
Prevalence of postpartum psychiatric conditions within each mode of delivery category. OVD, operative vaginal delivery.

Among the entire cohort, 9.8% had postpartum psychiatric diagnoses: 6.6% developed depression, 3.6% developed anxiety, 0.8% developed posttraumatic stress, and 0.2% developed a serious psychiatric condition (Fig. 2). In analyses both with and without adjustment for confounders, compared with people with SVDs (9.2%), the incidence of any psychiatric diagnosis in the first 6 months after delivery was increased in individuals who underwent planned cesarean deliveries (11.4%, adjusted RR 1.19, 95% CI, 1.17–1.21), unplanned cesarean deliveries without OVD attempt (10.8%, adjusted RR 1.16, 95% CI, 1.14–1.18), and unplanned cesarean deliveries with failed OVD attempt (10.9%, adjusted RR 1.26, 95% CI, 1.18–1.35 (Table 2 and Appendix 4, available online at http://links.lww.com/AOG/E671), but not in those with successful OVD (9.2% vs 9.2%, adjusted RR 1.00, 95% CI, 0.97–1.03).

When we examined individual psychiatric condition, all diagnoses were higher among people with planned cesarean deliveries or unplanned cesarean deliveries without OVD attempt compared with those with SVDs (Table 2). Similarly, those who had cesarean delivery after failed OVD had increased risk of depression, anxiety, and posttraumatic stress. Other severe psychiatric conditions (bipolar spectrum disorders, schizophrenia spectrum disorders, psychotic disorders, delusional disorders, and personality disorders) were too rare to assess.

**Fig. 3.**
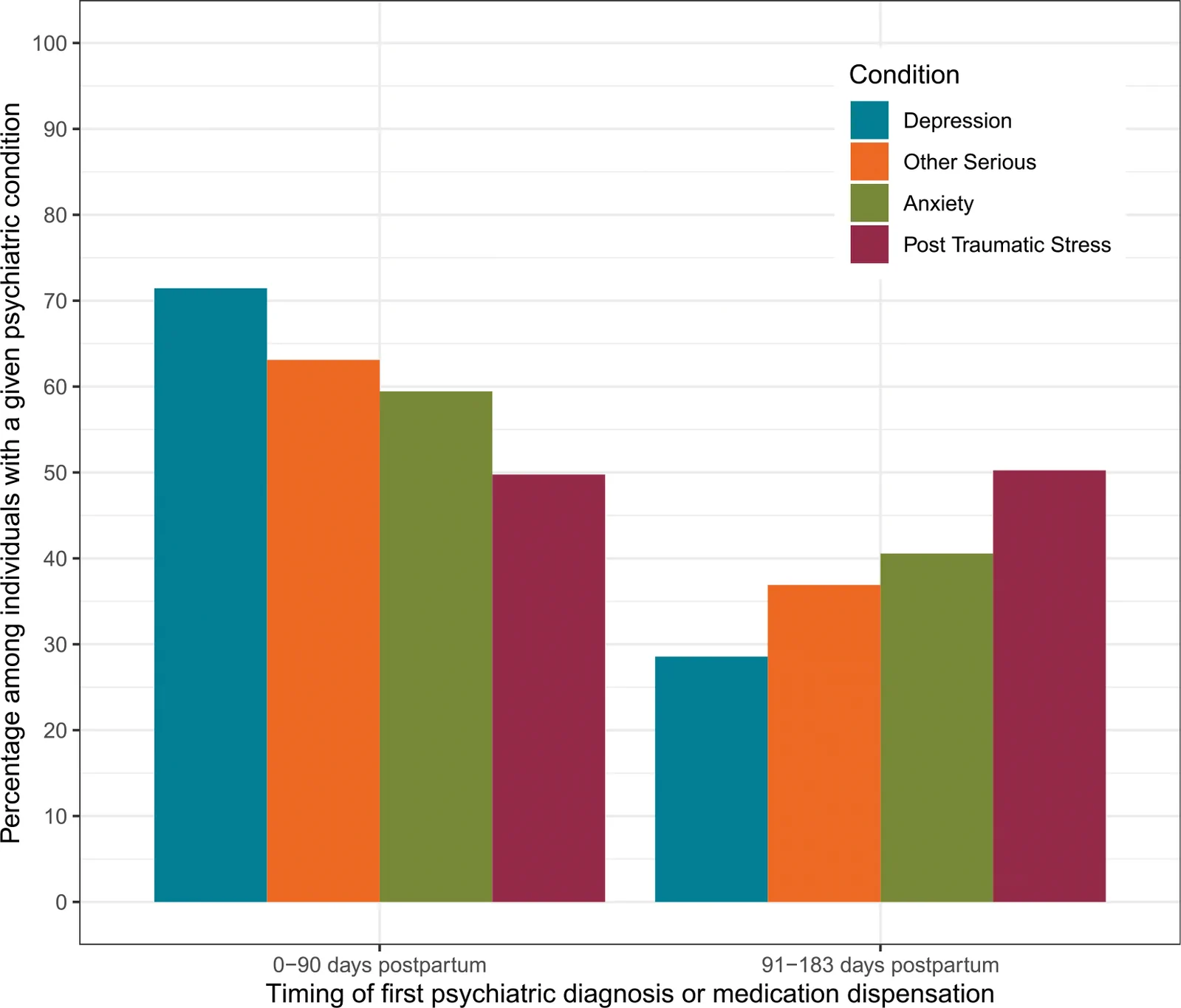
Timing of diagnosis of psychiatric condition between two postpartum timepoints.

Findings were similar in sensitivity analyses using generalized estimating equation models accounting for clustering of births to one person (Appendix 5, available online at http://links.lww.com/AOG/E671). Findings were also similar for the outcome of post-traumatic stress when restricted to ICD-10-CM (adjusted RR 1.45, 95% CI, 1.00–2.10 vs adjusted RR 1.40, 95% CI, 1.04–1.89) (Appendix 6, available online at http://links.lww.com/AOG/E671). In post hoc analysis, the majority of diagnoses of depression, anxiety, and other serious psychiatric conditions occurred in the 0–90 days after delivery. Posttraumatic stress emerged at a similar rate between 0–90 and 91–180 days after delivery (Fig. 3).

## DISCUSSION

In this study of nearly 1 million singleton pregnancies in individuals across the United States, new postpartum psychiatric conditions were more frequently diagnosed in those with cesarean births regardless of whether they were planned or unplanned and with or without an OVD attempt. In contrast, successful OVDs were not associated with postpartum psychiatric conditions. These results reinforce the current clinical practice of offering OVD for appropriate candidates and underscore the potential need for additional mental health surveillance for individuals who undergo cesarean delivery in any context.

Our study offers a comprehensive comparison of incident postpartum psychiatric conditions across all modes of delivery, specifically those that may be considered traumatic by patients. Results extend previously noted associations between unplanned cesarean delivery and postpartum psychiatric conditions by establishing that the mental health risks associated with cesarean delivery go beyond unplanned cesarean and depression alone.^2,23,24^ Findings suggest that the experience of cesarean birth itself, rather than only the emergency or unanticipated nature of the procedure, may contribute to the elevated psychiatric risk. The physical and emotional demands of recovering from major surgery while caring for a newborn, in addition to the potential deviations from patient expectations for vaginal delivery,^6,23,24^ may underlie these findings. The acute stress of emergency decision making that precedes unplanned cesarean delivery may further exacerbate patient fear, helplessness, or trauma, all of which may drive postpartum psychiatric conditions.^25,26^ This highlights the importance of monitoring and providing psychiatric support after cesarean delivery in the postpartum period and the need for tailored management strategies to identify and treat possible birth trauma related to these interventions.

This study also uniquely recognizes failed OVDs as a distinct mental health risk exposure. Prior studies had contradictory findings—Dekel et al^2^ reported higher psychological distress after successful OVD compared with SVD but Patel et al^12^ found no difference—yet neither study analyzed failed OVDs resulting in cesarean delivery separately from other delivery groups. By separating groups, we found that, unlike failed OVDs or unplanned cesarean deliveries, successful OVDs were not associated with an increased risk of postpartum psychiatric conditions. Because successful OVDs may preserve elements of a person’s birth goals, they may be perceived as less traumatic, potentially offering a degree of psychological protection in the postpartum period. Despite the known physical—and now mental—health benefits of a successful OVD in contrast to its alternative (a second-stage cesarean delivery), the use of operative deliveries has continued to decline in the United States.^27^ Reduced use may reflect diminished clinician comfort and proficiency with OVD, and our findings cannot directly determine the extent to which OVD could substitute for unplanned cesarean deliveries. These considerations highlight the need for nuanced evaluation of both maternal and neonatal outcomes—both psychiatric and physical—when weighing delivery approaches.

In contrast to prior studies reporting prevalence rates of postpartum psychiatric conditions of approximately 20%^5^ up to 12 months postpartum, we identified a lower overall prevalence of 9% within the first 6 months. One explanation is that our study focused on incident psychiatric diagnosis in the postpartum period, so exclusion of people with preexisting psychiatric conditions may be contributing. In addition, our reliance on ICD coding may capture only the most severe cases. The Merative^™^ MarketScan^®^ Commercial Database includes only privately insured patients, and previous research has shown that individuals with public insurance such as Medicaid experience higher rates of postpartum depression.^28^

Although our study highlights important associations, further research is needed to clarify the underlying mechanisms and to identify potential protective factors and modifiable risk factors. Postpartum psychiatric conditions are likely influenced by a range of downstream factors independently of mode of delivery that warrant further investigation such as neonatal outcomes, maternal complications, and psychosocial determinants. Future studies that incorporate patient perspectives and examine moderating variables may deepen our understanding of why cesarean births are associated with higher rates of postpartum psychiatric conditions. This study did not evaluate long-term mental health beyond 6 months postpartum. Future research is needed to understand how psychiatric conditions evolve over time after childbirth, particularly across distinct, potentially traumatic birth experiences.

Our study has several strengths, including the large sample size representative of the geographic diversity of the United States, which allowed us to evaluate a rare exposure (OVD attempts) to make unique contributions to the literature. In addition, the large sample size facilitated analyses of distinct psychiatric conditions, highlighting disorders beyond depression.

This study also has several limitations. The study population consisted only of individuals who had commercial insurance and received health care for psychiatric symptoms. This could underestimate the potential effects of traumatic delivery mode on postpartum mental health. Our study is additionally limited by the use of ICD codes, which are subject to misclassification, underascertainment, and temporal changes in screening, referral, and diagnostic practices over the study period. Specifically, assessment of unplanned, intrapartum cesarean births, particularly those in the second stage, is challenging because of the lack of well-defined corresponding codes to distinguish planned from unplanned cesarean deliveries. We addressed this by developing an algorithm to exclude conditions for which cesarean deliveries typically are planned.^16^ Despite these attempts to classify mode of delivery as accurately as possible, the distinction between planned and unplanned cesarean delivery remains limited by available data. In addition, the possibility of unmeasured confounding should be considered. This study included only patients with continuous health care encounters through 6 months postpartum, representing an idealized level of prenatal and postnatal care, which may limit generalizability. Finally, these results may not be generalizable to individuals with preexisting psychiatric conditions.

In this large, contemporary cohort, we found that cesarean births—whether planned or preceded by an OVD attempt—were independently associated with new postpartum psychiatric conditions up to 6 months postpartum. In contrast, successful OVDs were not associated with new psychiatric conditions. Strategies aimed at increasing OVD success rates may also contribute to better postpartum psychiatric outcomes. Maternal psychiatric conditions are common, and understanding and mitigating the psychological toll of childbirth interventions is a critical priority in modern perinatal care.

## Supplementary Material

Appendices 1-6

## Figures and Tables

**Table 1.** Clinical Characteristics and Birth Outcomes of Individuals With Term Singleton Live Births Who Underwent Trial of Labor in the United States Between 2008 and 2022 (N = 934,524)

Characteristic	SVD (n = 618,594)	Successful OVD (n=42,860)	Planned Cesarean (n=130,571)	Unplanned Cesarean, No OVD Attempt (n=135,808)	Cesarean After Failed OVD (n = 6,691)	*P* *

Sociodemographic characteristics						
Age (y)						<.001
Younger than 25	36,793 (6)	2,906 (7)	3,826 (3)	7,085 (5)	314 (5)	
25–29	186,915 (30)	14,435 (34)	28,203 (22)	37,784 (28)	1,907 (29)	
30–34	259,048 (42)	17,098 (40)	55,817 (43)	55,280 (41)	2,761 (41)	
35–39	115,698 (19)	7,041 (16)	34,256 (26)	28,476 (21)	1,378 (21)	
40 or older	20,140 (3)	1,380 (3)	8,469 (7)	7,183 (5)	331 (5)	
U.S. region						<.001
North Central	153,451 (25)	11,393 (27)	28,081 (22)	30,005 (22)	2,122 (32)	
Northeast	108,225 (17)	6,914 (16)	20,900 (16)	24,330 (18)	907 (14)	
South	244,383 (40)	16,664 (39)	62,233 (48)	60,600 (45)	2,439 (36)	
West	105,324 (17)	7,391 (17)	17,899 (14)	19,184 (14)	1,079 (16)	
Delivery year						<.001
2008–2012	278,545 (45)	21,050 (49)	47,210 (36)	62,043 (46)	3,916 (59)	
2013–2017	207,979 (34)	13,518 (32)	46,375 (36)	45,867 (34)	2,186 (3)	
2018–2022	132,070 (21)	8,292 (19)	36,989 (28)	27,898 (21)	589 (8.8)	
Clinical characteristics and birth outcomes						
BMI 40 or higher	8,028 (1)	435 (1)	4,169 (3)	4,513 (3)	164 (3)	<.001
Substance use during pregnancy^†^	3,886 (1)	292 (1)	924 (1)	1,182 (1)	43 (1)	<.001
Chronic hypertension	20,967 (3)	1,508 (4)	7,542 (6)	8,575 (6)	437 (7)	<.001
Diabetes (type 1 or 2)	16,408 (3)	1,206 (3)	5,688 (4)	6,385 (5)	371 (6)	<.001
Gestational hypertension or preeclampsia without severe features	58,055 (9)	4,611 (11)	15,251 (12)	20,694 (15)	848 (13)	<.001
Preeclampsia with severe features	7,691 (1)	655 (2)	3,332 (3)	4,467 (3)	161 (2)	<.001
Gestational age at delivery (wk)	39.0±0.6	39.0±0.5	38.9±0.5	39.0±0.6	39.0±0.4	<.001
Induction of labor	121,752 (20)	9,300 (22)	3,503 (3)	21,961 (16)	845 (13)	<.001
Postpartum hemorrhage	19,173 (3)	1,719 (4)	2,227 (2)	3,194 (2)	169 (3)	<.001
Intra-amniotic infection	13,551 (2)	2,387 (6)	1,859 (2)	8,471 (6)	293 (4)	<.001
Previous cesarean delivery	8,789 (13)	775 (1)	36,519 (54)	20,181 (30)	1,422 (2)	<.001
Severe maternal morbidity^‡^	5,039 (1)	589 (1)	2,293 (2)	2,947 (2)	154 (2)	<.001
Severe unexpected complications in term newborns^§^	11,220 (2)	1,773 (4)	2,244 (2)	4,829 (4)	227 (3)	<.001

SVD, spontaneous vaginal delivery; OVD, operative vaginal delivery; BMI, body mass index.

Data are n (column %) or mean±SD unless otherwise specified.

* Categorical variables were compared with the Pearson χ^2^ test, and continuous variables were compared with the Kruskal–Wallis rank-sum test.

† Includes alcohol, tobacco, hallucinogens, cocaine, amphetamines, inhalants, or other unspecified drug use.

‡ Severe maternal morbidity index, as defined by the Centers for Disease Control and Prevention; 21 indicators with associated International Classification of Diseases codes, including transfusion.

§ Includes any of the following: severe birth trauma, severe hypoxia or asphyxia, severe shock and resuscitation, neonatal severe respiratory complications, neonatal severe infection, neonatal severe neurologic complications, neonatal severe septicemia, severe shock and resuscitation procedures, neonatal severe respiratory procedures, and neonatal severe neurologic procedures.

**Table 2.** Adjusted Risk Ratios for New-Onset Postpartum Psychiatric Conditions by Mode of Delivery Among Individuals With Term Singleton Live Births Undergoing Trial of Labor in a Commercial Claims Database (N = 934,524)

		Successful OVD (n=42,860)		Planned Cesarean (n=130,571)	
Psychiatric Condition	SVD (Ref) (n=618,594)	n (%)	aRR (95% CI)*	n (%)	aRR (95% CI)^†^

Any	57,191 (9.2)	3,936 (9.2)	1.00 (0.97–1.03)	14,957 (11.4)	1.19 (1.17–1.21)
Depression	38,550 (6.2)	2,693 (6.3)	1.00 (0.96–1.04)	9,787 (7.5)	1.19 (1.16–1.22)
Anxiety	20,958 (3.4)	1,372 (3.2)	0.97 (0.92–1.03)	5,691 (4.4)	1.18 (1.14–1.21)
Posttraumatic stress	4,559 (0.7)	324 (0.8)	1.09 (0.97–1.22)	1,351 (1.0)	1.12 (1.06–1.19)
Serious mental illness^‡^	1,239 (0.2)	82 (0.2)	0.93 (0.74–1.16)	311 (0.2)	1.25 (1.10–1.42)

	Unplanned Cesarean With No OVD Attempt (n = 135,808)		Cesarean After Failed OVD (n=6,691)	
Psychiatric Condition	n (%)	aRR (95% CI)*	n (%)	aRR (95% CI)^†^

Any	14,747 (10.8)	1.16 (1.14–1.18)	731 (10.9)	1.26 (1.18–1.35)
Depression	9,983 (7.4)	1.17 (1.14–1.19)	509 (7.6)	1.23 (1.13–1.34)
Anxiety	5,424 (4.0)	1.18 (1.14–1.21)	249 (3.7)	1.30 (1.15–1.47)
Posttraumatic stress	1,111 (0.8)	1.10 (1.03–1.18)	43 (0.6)	1.40 (1.04–1.89)
Serious mental illness^‡^	333 (0.2)	1.19 (1.06–1.35)	16 (0.2)	1.14 (0.69–1.86)

SVD, spontaneous vaginal delivery; OVD, operative vaginal delivery; aRR, adjusted risk ratio.

Data are n (column %) unless otherwise specified.

* Models adjusted for maternal age, region, delivery year, class III obesity, substance use, gestational age at delivery, chronic hypertension, and prepregnancy diabetes (type 1 or 2).

† Poisson regression model with robust SEs.

‡ Includes bipolar spectrum disorders, schizophrenia spectrum disorders, psychotic disorders, delusional disorders, and personality disorders.
