# Supplementary material for: Association Between Delivery Mode and Postpartum Psychiatric Conditions
Source: Obstet Gynecol. 2026 May 21;148(4):e257–65. doi: 10.1097/AOG.0000000000006321 (PMC13439854; doi:10.1097/AOG.0000000000006321)
Supplement: Supplementary file 1 [file ong-148-e257-s001.pdf]

Appendix 1. ICD diagnosis codes used to classify independent and dependent variables.

Diagnosis	ICD-9	ICD-10
Depressive Disorders	"2962" "2963" "2968" "3004" "3091" "311"	"F320" "F321" "F323" "F324" "F325" "F328" "F329" "F330" "F331" "F332" "F333" "F334" "F338" "F339" "F341" "F530"
Anxiety Disorders	"2938" "3000" "3002" "3003"	"F410" "F411" "F412" "F413" "F418" "F419" "F400" "F401" "F402" "F408" "F409"
Post traumatic stress symptoms	"3098"	"F430" "F431" "F432" "F438" "F439"
Other serious mental health condition	"2960" "2961" "2964" "2965" "2966" "2967" "2968" "2969" "2950" "2951" "2952" "2953" "2954" "2955" "2956" "2957" "2958" "2959"	"F201" "F202" "F203" "F205" "F208" "F208" "F209" "F22" "F23" "F24" "F250" "F251" "F258" "F259" "F28" "F29" "F310" "F311" "F312" "F313" "F314" "F315" "F316" "F317" "F318" "F319" "F30.1" "F302" "F303" "F304" "F308" "F309" "F600" "F601" "F602" "F603" "F604" "F605" "F606" "F607" "F6081" "F6089" "F609" "F531"
Vacuum extraction	"7271" "7279"	"O814" "10D07Z6"
Forceps extraction	"720" "721"	"O810" "O813" "10D07Z3"
Other instrumental delivery	"6607" "6695" "728" "729"	"O665"

Kruger SL, Perlman N, Sherwin EB, Leggett CB, El-Sayed Y, Lyell DJ, et al. Association between delivery mode and postpartum psychiatric conditions. Obstet Gynecol 2026;147.
The authors provided this information as a supplement to their article.
©2026 American College of Obstetricians & Gynecologists.

Appendix 2. Antidepressant medications used to define depression

Tricyclic Antidepressants	Selective Serotonin Reuptake Inhibitors	Neurosteroid Antidepressant
Amitriptyline<br>Amoxapine<br>Desipramine<br>Doxepin<br>Imipramine<br>Nortriptyline<br>Protriptyline<br>Trimipramine<br>Clomipramine	Citalopram<br>Escitalopram<br>Fluoxetine<br>Fluvoxamine<br>Paroxetine<br>Sertraline<br>Vilazodone	Zuranolone<br>Brexanolone

Kruger SL, Perlman N, Sherwin EB, Leggett CB, El-Sayed Y, Lyell DJ, et al. Association between delivery mode and postpartum psychiatric conditions. Obstet Gynecol 2026;147.
The authors provided this information as a supplement to their article.
©2026 American College of Obstetricians & Gynecologists.

Appendix 3. ICD diagnosis codes used to identify comorbidities

Variable	ICD-9	ICD-10
Class 3 obesity, BMI ≥ 40 kg/m^2	"27801"	"Z684" "E6601" "E662"
Diabetes mellitus types 1 and 2	"250" "6480"	"E08" "E09" "E10" "E11" "E12" "E13" "O240" "O241" "O243" "O248" "O249" "Z794"
Chronic hypertension	"401" "6420" "6427"	"O100" "O11" "I10"
Gestational hypertension or mild preeclampsia	"6423" "6424"	"O13" "O140" "O149"
Severe preeclampsia	"6425" "6427"	"O11" "O141" "O142"
Substance use*	"64830" "64832" "64833" "291" "292" "303" "3041" "3042" "3043" "3044" "3045" "3046" "3048" "3049" "3050" "3051" "3052" "3053" "3056" "3057" "3059"	"O9931" "O9932" "F10" "F12" "F13" "F14" "F15" "F16" "F17" "F18" "F19"
Postpartum hemorrhage	"6660" "6661" "6662"	"O720" "O721" "O722"
Intraamniotic infection	"6584"	"O411"
Labor complication†	"653" "6590" "6591" "6592" "6593" "6597" "660" "661" "662" "663" "664" "665" "6695" "6696" "6697"	"O33" "O61" "O62" "O63" "O64" "O65" "O66" "O69" "O70" "O71" "O752" "O753" "O7581" "O7582" "O76" "O77"
Induction of labor†	"7301" "731" "734"	"3E033VJ" "0U7C7ZZ" "0U7C7DZ" "3E0P7GC"
Multifetal gestation	"651" "V272" "V273" "V274" "V275" "V276" "V277"	"O30" "O31" "O632" "Z372" "Z373" "Z374" "Z375" "Z376" "Z377"
Fetal malpresentation	"6522" "6523" "6524" "6520" "6527" "6528" "6529" "6696"	"O32" "O642" "O643" "O644" "O645" "O648" "O649" "0830" "0831"
Vasa previa	"6635"	"0694"
Placenta previa	"6410" "6411"	"O4400" "O4403" "O4410" "O4413" "O4420" "O4423" "O4440" "O4443" "O4450" "O4453" "O4430" "O4433"
Mid or high forceps	"772x" "723x"	"O811" "10D07Z4" "10D07Z5"
Rotational forceps	"724"	"O812"
Breech extraction or Forceps for aftercoming head	"725x" "726"	"O830" "O831"

* Substance use included alcohol, tobacco, hallucinogens, cocaine, amphetamines, inhalants, or other nonspecified drug use

† The presence of any ICD-9-CM, ICD-10-CM, or CPT code within these two categories, along with a cesarean delivery procedure code, was used to identify unplanned or intrapartum cesarean deliveries.

©2026 American College of Obstetricians & Gynecologists.

Appendix 4. Risk ratios for new-onset postpartum psychiatric conditions by mode of delivery among term, singleton, livebirths undergoing a trial of labor in a commercial claims database (N=934,524)

	**Successful OVD**	**Planned Cesarean Delivery**	**Unplanned cesarean with no OVD attempt**	**Cesarean after failed OVD**[*]
	RR [†] (95% CI)	RR (95% CI)	RR (95% CI)	RR (95% CI)
**Any Mental Health Diagnosis**	0.99 (0.96, 1.02)	1.24 (1.22, 1.26)	1.17 (1.15, 1.19)	1.18 (1.10, 1.27)
**Depression**	1.01 (0.97, 1.05)	1.20 (1.18, 1.23)	1.18 (1.15, 1.20)	1.22 (1.12, 1.33)
**Anxiety**	0.94 (0.90, 1.00)	1.29 (1.25, 1.32)	1.18 (1.14, 1.21)	1.10 (0.97, 1.24)
**Post Traumatic Stress**	1.03 (0.92, 1.15)	1.40 (1.32, 1.49)	1.11 (1.04, 1.18)	0.87 (0.65, 1.18)
**Serious Mental Illness** [‡]	0.96 (0.76, 1.19)	1.19 (1.05, 1.35)	1.22 (1.08, 1.38)	1.19 (0.73, 1.95)

[*]Abbreviations: RR = risk ratio, OVD = Operative Vaginal Delivery
[†] Risk ratio of postpartum mental condition in comparison to spontaneous vaginal delivery (referent group)
[‡] Serious mental illnesses included bipolar spectrum disorders, schizophrenia spectrum disorders, psychotic disorders, delusional disorders and personality disorders

Kruger SL, Perlman N, Sherwin EB, Leggett CB, El-Sayed Y, Lyell DJ, et al. Association between delivery mode and postpartum psychiatric conditions. Obstet Gynecol 2026;147.
The authors provided this information as a supplement to their article.
©2026 American College of Obstetricians & Gynecologists.

Appendix 5: Sensitivity analysis of postpartum mental conditions by mode of delivery, using Generalized Estimating Equations (GEE) to account for clustering of multiple births per person.

	Successful OVD*		Planned Cesarean Delivery		Unplanned cesarean delivery with no OVD attempt		Cesarean delivery after failed OVD	
	RR*† (95% CI)	aRR*† (95% CI)	RR (95% CI)	aRR (95% CI)	aRR (95% CI)	aRR (95% CI)	RR (95% CI)	aRR (95% CI)
Any Mental Condition	0.99 (0.96, 1.02)	1.00 (0.97, 1.03)	1.24 (1.22, 1.26)	1.18 (1.16, 1.20)	1.17 (1.15, 1.19)	1.16 (1.14, 1.18)	1.18 (1.10, 1.26)	1.26 (1.17, 1.35)
Depression	1.00 (0.96, 1.04)	1.00 (0.96, 1.03)	1.20 (1.18, 1.23)	1.19 (1.16, 1.21)	1.17 (1.15, 1.20)	1.16 (1.14, 1.19)	1.22 (1.12, 1.32)	1.22 (1.12, 1.32)
Anxiety	0.94 (0.89, 0.99)	0.97 (0.92, 1.02)	1.28 (1.25, 1.32)	1.17 (1.14, 1.21)	1.17 (1.14, 1.21)	1.17 (1.14, 1.21)	1.10 (0.97, 1.24)	1.30 (1.15, 1.47)
Post Traumatic Stress	1.02 (0.92, 1.15)	1.09 (0.97, 1.22)	1.40 (1.32, 1.49)	1.12 (1.06, 1.19)	1.11 (1.04, 1.18)	1.10 (1.03, 1.18)	0.87 (0.65, 1.18)	1.40 (1.04, 1.89)
Serious Mental Illness ‡	0.96 (0.76, 1.19)	0.93 (0.74, 1.16)	1.19 (1.05, 1.34)	1.25 (1.10, 1.42)	1.22 (1.08, 1.38)	1.19 (1.05, 1.35)	1.19 (0.73, 1.95)	1.14 (0.70, 1.86)

*Abbreviations: OVD = Operative Vaginal Delivery, RR = Risk Ratio, aRR = Adjusted Risk Ratio.
† Risk ratio and adjusted risk ratio compared to referent group of spontaneous vaginal delivery
‡ Serious mental illnesses included bipolar spectrum disorders, schizophrenia spectrum disorders, psychotic disorders, delusional disorders and personality disorders

Kruger SL, Perlman N, Sherwin EB, Leggett CB, El-Sayed Y, Lyell DJ, et al. Association between delivery mode and postpartum psychiatric conditions. Obstet Gynecol 2026;147.
The authors provided this information as a supplement to their article.
©2026 American College of Obstetricians & Gynecologists.

Appendix 6: Sensitivity analysis of post-traumatic stress during the ICD-10 years only (2016-2022)

	Successful OVD*		Planned Cesarean Delivery		Unplanned cesarean delivery with no OVD attempt		Cesarean delivery after failed OVD	
	RR*† (95% CI)	aRR*† (95% CI)	RR (95% CI)	aRR (95% CI)	aRR (95% CI)	aRR (95% CI)	RR (95% CI)	aRR (95% CI)
Post Traumatic Stress	1.12 (0.99, 1.26)	1.10 (0.97, 1.24)	1.08 (1.01, 1.15)	1.07 (1.00, 1.14)	1.14 (1.07, 1.23)	1.11 (1.03, 1.19)	1.53 (1.05, 2.22)	1.45 (1.00, 2.10)

*Abbreviations: OVD = Operative Vaginal Delivery, RR = Risk Ratio, aRR = Adjusted Risk Ratio.
† Risk ratio and adjusted risk ratio compared to referent group of spontaneous vaginal delivery

The authors provided this information as a supplement to their article.
©2026 American College of Obstetricians & Gynecologists.
