# Supplementary material for: Association Between Delivery Mode and Postpartum Psychiatric Conditions
Source: Obstet Gynecol. 2026 May 21;148(4):e257–65. doi: 10.1097/AOG.0000000000006321 (PMC13439854; doi:10.1097/AOG.0000000000006321)
Supplement: Supplementary file 2 [file ong-148-e257-s002.pdf]

NOTICE: This document contains correspondence generated during peer review and subsequent revisions but before transmittal to production for composition and copyediting:

- Comments from the reviewers and editors (email to author requesting revisions)
- Response from the author (cover letter submitted with revised manuscript)*

**The corresponding author has opted to make this information publicly available.*

Personal or nonessential information may be redacted at the editor's discretion.

Questions about these materials may be directed to the *Obstetrics & Gynecology* editorial office: obgyn@greenjournal.org.

**Date:**	03/10/2026
**To:**	"Danielle M. Panelli" [redacted]
**From:**	"The Green Journal" em@greenjournal.org
**Subject:**	Your Submission ONG-26-214

03/10/2026

RE: ONG-26-214: Association between delivery mode and postpartum psychiatric conditions: A national cohort study

Dear Dr. Panelli:

Thank you for sending us your work for consideration for publication in Obstetrics & Gynecology. Your manuscript has been reviewed by the Editorial Board and by special expert referees. The Editors would like to invite you to submit a revised version for further consideration.

If you wish to revise your manuscript, please read the following comments submitted by the reviewers and Editors. Each point raised requires a response, by either revising your manuscript or making a clear argument as to why no revision is needed in the cover letter.

To facilitate our review, we prefer that the cover letter you submit with your revised manuscript include each reviewer and Editor comment below, followed by your response. That is, a point-by-point response is required to each of the EDITOR COMMENTS (if applicable), REVIEWER COMMENTS, and STATISTICAL EDITOR COMMENTS (if applicable) below. The revised manuscript should indicate the position of all changes made. Please use the "track changes" feature in your document (do not use strikethrough or underline formatting). Upload the tracked-changes version when you submit your revised manuscript.

Your submission will be maintained in active status for 21 days from the date of this letter. If we have not heard from you by 03/31/2026, we will assume you wish to withdraw the manuscript from further consideration.

EDITOR COMMENTS:

Please note the following:

* Help us reduce the number of queries we add to your manuscript after it is revised by reading the Revision Checklist at https://journals.lww.com/greenjournal/Documents/RevisionChecklist_Authors.pdf and making the applicable edits to your manuscript.

* As of January 2024, only certain article types will appear in the print version of the journal. All accepted articles will continue to publish online. All articles will be indexed in PubMed as an official article of Obstetrics & Gynecology. Additional information is available in the Instructions for Authors (https://journals.lww.com/greenjournal/Pages/InformationforAuthors.aspx#II).

* Figure 1: Please check n-values for accuracy.
* Figures 2 and 3: Please upload a higher resolution version of these figures.

REVIEWER COMMENTS:

Reviewer #1:
This is a cross sectional analysis using the MarketScan Commercial Database in which investigators sought to explore the relationship between mode of delivery and postpartum incident psychiatric disorders. The authors found that compared to spontaneous vaginal births, any cesarean (planned, unplanned or following failed operative delivery) was associated with an increased risk of postpartum psychiatric conditions.

The authors should be applauded for recognizing the increasing importance of traumatic birth and the need to enhance the delivery of trauma-informed obstetric care (e.g. Froeliger A et al, AJOG 2026; Baldini V et al, Psychiatr Q).

However, the study suffers methodologically as the authors stated primary objective was to assess the association of operative delivery on postpartum mental health conditions yet perform analyses comparing 5 different exposures, raising

the concern for multicollinearity and lack of conceptual coherence. For example, rates of hypertensive disorder, labor induction and IAI appear to differ across groups and may be collinear or confounding. The PC06 measure does not differ, but was there a difference in NICU or special care nursery admissions? Did the authors consider sensitivity analyses to model these effects? This study evaluates a timely and clinically relevant question, however the methodological framework is insufficient to draw more than casually exploratory observations.

Reviewer #2: This is a large national cohort study using ICD codes to study the connection between mode of delivery and new diagnosis of depression, post-traumatic stress, and other serious mental illnesses or anti-depressive medication prescription within 6 months of delivery. Nearly 1 million births were included, making it one of the larger studies of its kind and undoubtedly of interest to obstetric providers as they identify patients at risk for mental health diagnoses. It is a very well-written manuscript and quite topical. I enjoyed reading and reviewing it, and I feel it adds something significant to the body of maternal mental health literature. In order to further improve their work, the authors might consider the following:

Introduction:
1. Throughout the body of the manuscript and beginning at line 58, you refer to "post-traumatic stress". Unlike the other conditions mentioned, my understanding is that this can refer to temporary symptoms rather than a diagnosis based on DSM-V criteria such as Post-Traumatic Stress Disorder. I don't think it's wrong to include post-traumatic stress as a maternal mental health outcome of study, but I would recommend further defining it in the introduction and making the distinction between this condition and an actual PTSD diagnosis.

2. For lines 72-82, if you are trying to make the argument that perhaps obstetricians are shying away from OVD because of concerns over creating trauma, I think the comparison between prevalence of OVD in the US versus the UK and other countries can be left out. I am not sure what it adds to this argument.

Materials and Methods:
1. In line 102, why were "unusual or rare forceps deliveries" excluded? It would be nice to see this similarly addressed to how the other inclusion/exclusion criteria are explained.

2. I am a little confused by lines 116-119. Were only the cesareans with an accompanying code for placenta prevue, vasa previa, and fetal malpresentation included in the planned cesarean group? I think this could be reworded to read something like "the planned cesarean group was comprised of patients who had both a code for cesarean and a code for a diagnosis contraindicating vaginal delivery, including placenta prevue, vasa previa, and fetal malpresentation". You could also consider including patients with a prior classical.

3. I am not sure if I would include BPD, schizophrenia, and personality disorders as part of your composite outcome. These disorders, to my understanding, are unlikely to emerge as a direct result of experiencing traumatic birth and perhaps more likely to be unmasked by the stressors accompanying new parenthood (like lack of sleep). They are also quite uncommon.

4. Did you consider adding pregnancy induced hypertensive disorders to your covariates, or postpartum hemorrhage, both of which can be independent causes of trauma? They might be worth including as potential confounders.

Results:
No suggestions

Discussion:
1. For line 193, my understanding is that you included cesareans formed for malpresentation- if this is the case, I would recommend removing "vertex-presenting" from this line. If I somehow misunderstood and you did exclude patients who had a planned CS for malpresentation, this bears explaining.

2. For lines 243-244, is there a reason you suspect that social support (presumably decreased support) underlies the higher rate of mental health diagnoses seen in your study? If not, I would remove the hyphenated clause. If so, I would flesh this out more. It's been well-demonstrated that limited social support is a risk factor for adverse mental health outcomes, but I am not sure it's plausible as a moderating variable that would contribute to the higher rates of mental health disorder seen in patients undergoing cesarean.

3. I would recommend in some way addressing the way that you handled TOLAC (which could simply be to mention that you were limited by the information included in the ICD codes and were likely not able to accurately separate TOLAC cases, which might have their own inherent risk factors for postpartum mental health disorders) and the way that you handled the COVID-19 pandemic (which it appears you did by controlling for the delivery year) in your discussion somewhere.

Reviewer #3: The authors have presented a very large study showing association between mode of delivery and psychiatric illness post partum.
The limitation that the sample although huge consists of only women who have private insurance and sought help for the

psychiatric symptoms is stated.
The sample depends on accurate coding and this may decrease generalisability.

STATISTICAL EDITOR COMMENTS:
Lines 102-104. Is the exclusion of these types of operative deliveries normally done in the literature? How many were excluded due to this reason? It may be worth adding sensitivity analyses with these participants included, they may be more likely to experience the outcome.

Lines 117-118. This doesn't appear to be an exhaustive list of the criteria for planned cesarean deliveries. If there was a cesarean delivery without a trial of labor and no placenta previa, etc. were they excluded? The exclusion criteria may need to be updated.

Lines 165-170 and Table 1. Statistical tests should be added to the analytical plan to describe significant differences in characteristics between exposure groups.

Lines 171-173. Data for the entire cohort is not presented in Figure 2.

Lines 175-177. Adjusted estimates are presented, the text should state that the results were obtained after adjusting for covariates.

Line 177. The aRR should be 1.26.

Table 1. Include the covariates in the footnote.

Lines 187-189. This data is not in the table. If elsewhere, please state the location.

--
Sincerely,

The Editors of Obstetrics & Gynecology

In compliance with data protection regulations, you may request that we remove your personal registration details at any time. (Use the following URL: https://www.editorialmanager.com/ong/login.asp?a=r). Please contact the publication office if you have any questions.

Stanford MEDICINE | DEPARTMENT OF OBSTETRICS AND GYNECOLOGY
STANFORD UNIVERSITY SCHOOL OF MEDICINE

March 30, 2026

Dear Editors of *Obstetrics & Gynecology*,

We are delighted to submit revisions to our manuscript entitled, "*Association between delivery mode and postpartum psychiatric conditions: A national cohort study"* for your ongoing review. We appreciate the Editor and Reviewer feedback, and have incorporated all suggestions in the attached point-by-point Response to Review. We agree that these changes have yielded a stronger manuscript. This abstract was presented at the 45th Annual Pregnancy Meeting of the Society for Maternal-Fetal Medicine, January 27 – February 1, 2025.

To summarize our study, amid growing recognition of the profound impact that childbirth experiences can have on maternal mental health, our manuscript addresses a critical gap in understanding the psychiatric consequences of all delivery modes. Using a large, national cohort of over 900,000 commercially insured individuals with no prior psychiatric diagnoses, we examined the association between all modes of delivery—including spontaneous vaginal delivery, successful operative vaginal delivery (OVD), planned cesarean delivery, unplanned cesarean delivery without OVD attempt, and unplanned cesarean following failed OVD attempt—and incident postpartum psychiatric conditions by six months postpartum. A key methodological strength is the detailed stratification of delivery types, which allowed us to disentangle the mental health risks associated with failed versus successful OVDs—an area of nuance not captured in most prior research. We found that while planned and unplanned cesareans, with and without prior OVD attempt, were associated with elevated risk of postpartum psychiatric conditions, successful OVDs were not.

Our results suggest that not all delivery modes confer equal psychiatric risk. These insights underscore the importance of preserving OVD as an alternative to cesarean when clinically indicated and highlight the need for postpartum mental health surveillance following all cesarean deliveries. We believe these findings will be of strong interest to *Obstetrics & Gynecology's* readership and contribute meaningfully to the ongoing conversation about optimizing mental health outcomes in obstetric care.

This study was approved by the Stanford University Institutional Review Board. Each author has substantially contributed to study design, data analysis, and manuscript development. All authors approve this submission and report no conflict of interest. This manuscript is solely in submission to *Obstetrics & Gynecology*. We look forward to your response to our manuscript and welcome your feedback.

Sincerely,

[redacted]

Danielle Panelli, MD MS*

*Corresponding author and manuscript's guarantor

RESPONSE TO REVIEW

MANUSCRIPT ID: ONG-26-214
*Note: line references in Reviewer comments refer to the original manuscript, and line references in our responses below refer to the revised manuscript with all changes accepted.

EDITOR COMMENTS:

Please note the following:

* Help us reduce the number of queries we add to your manuscript after it is revised by reading the Revision Checklist at https://urldefense.com/v3/__https://journals.lww.com/greenjournal/Documents/RevisionChecklist_Authors.pdf__;!!G92We9drHetJ8EofZw!Yw_7955niuI8833h72cyuKyYjHZrlNQRG4O5xthRh7Fn_JjZqaA_PVDou42lEPKTeQIQ77Op72WPD0UtGecN$ and making the applicable edits to your manuscript.

Thank you - I have reviewed the checklist and made the applicable edits.

* As of January 2024, only certain article types will appear in the print version of the journal. All accepted articles will continue to publish online. All articles will be indexed in PubMed as an official article of Obstetrics & Gynecology. Additional information is available in the Instructions for Authors (https://urldefense.com/v3/__https://journals.lww.com/greenjournal/Pages/InformationforAuthors.aspx*II__;Iw!!G92We9drHetJ8EofZw!Yw_7955niuI8833h72cyuKyYjHZrlNQRG4O5xthRh7Fn_JjZqaA_PVDou42lEPKTeQIQ77Op72WPD2OdsTgm$).

* Figure 1: Please check n-values for accuracy.

The numbers in Figure 1 have been checked for accuracy, with the revised figure shown below.

Figure 1. Study selection flow diagram

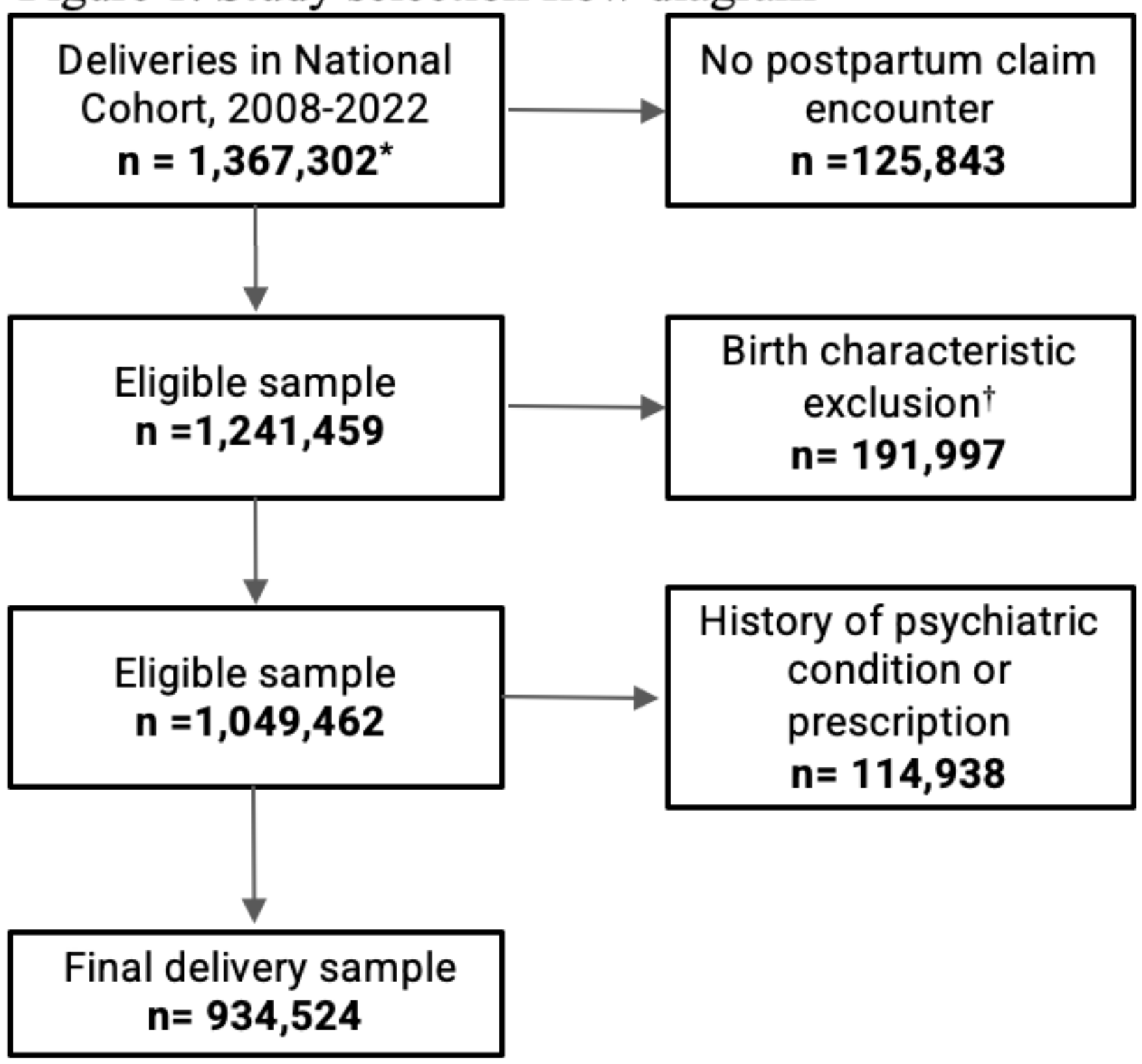

* Figures 2 and 3: Please upload a higher resolution version of these figures.

A higher resolution version of the figures has been uploaded with the resubmission.

REVIEWER COMMENTS:

Reviewer #1: This is a cross sectional analysis using the MarketScan Commercial Database in which investigators sought to explore the relationship between mode of delivery and postpartum incident psychiatric disorders. The authors found that compared to spontaneous vaginal births, any cesarean (planned, unplanned or following failed operative delivery) was associated with an increased risk of postpartum psychiatric conditions. The authors should be applauded for recognizing the increasing importance of traumatic birth and the need to enhance the delivery of trauma-informed obstetric care (e.g. Froeliger A et al, AJOG 2026; Baldini V et al, Psychiatr Q).

1. However, the study suffers methodologically as the authors stated primary objective was to assess the association of operative delivery on postpartum mental health conditions yet perform analyses comparing 5 different exposures, raising the concern for multicollinearity and lack of conceptual coherence. For example, rates of hypertensive disorder, labor induction and IAI

appear to differ across groups and may be collinear or confounding. The PC06 measure does not differ, but was there a difference in NICU or special care nursery admissions? Did the authors consider sensitivity analyses to model these effects? This study evaluates a timely and clinically relevant question, however the methodological framework is insufficient to draw more than casually exploratory observations.

We agree with the Reviewer that our objective was to evaluate associations between all possible modes of delivery (including operative vaginal delivery) and mental health conditions. We would like to clarify that we did not analyze 5 different exposures, but rather one exposure comprised of 5 mutually exclusive categories (4 indicator variables in a regression model). We did evaluate one singular composite primary outcome (mental health conditions), and then in separate models assessed individual diagnoses as separate outcomes. This modeling approach should minimize concerns regarding multicollinearity and conceptual coherence, and has been clarified in the Methods.

We also agree about the importance of selecting the right potential covariates for the model, and we wanted to ensure that every exposure group could, in theory, have the potential to experience the selected confounder (the positivity assumption in causal inference methods for observational data). For example, someone with a planned cesarean would not undergo a trial of labor and typically would be delivered prior to developing a labor complication (such as intra-amniotic infection). The causal framework we developed therefore included the following as potential confounders: age, region, delivery year, class III obesity (body mass index of ≥ 40 kg/m2) prior to delivery, substance use during pregnancy, gestational age at delivery, chronic hypertension, diabetes, and severe unexpected newborn complications (the PC06 measure referred to by the Reviewer). NICU admission was not available in our dataset. We agree that many other factors (in addition to delivery mode) may affect postpartum mental health, such as infant health complications and sleep deprivation, that are beyond the scope of the present study but would be important to assess in future work. To address this comment, additional edits have been made to the Methods and Discussion.

> Lines 110-114: The exposure was mode of delivery, modeled as one variable with five mutually exclusive categories. This variable was categorized as: spontaneous vaginal delivery (SVD), successful OVD with vacuum or forceps, planned cesarean, unplanned cesarean without OVD attempt, or cesarean after failed OVD.

> Lines 158-162: While there are other potential confounding factors that may affect postpartum mental health, we were intentional in selecting confounders that theoretically could affect every category within the mode of delivery exposure (e.g. planned cesareans would not typically experience complications related to labor, such as intraamniotic infection).

Reviewer #2: This is a large national cohort study using ICD codes to study the connection between mode of delivery and new diagnosis of depression, post-traumatic stress, and other serious mental illnesses or anti-depressive medication prescription within 6 months of delivery. Nearly 1 million births were included, making it one of the larger studies of its kind and undoubtedly of interest to obstetric providers as they identify patients at risk for mental health diagnoses. It is a very well-written manuscript and quite topical. I enjoyed reading and reviewing

it, and I feel it adds something significant to the body of maternal mental health literature. In order to further improve their work, the authors might consider the following:

1. Throughout the body of the manuscript and beginning at line 58, you refer to "post-traumatic stress". Unlike the other conditions mentioned, my understanding is that this can refer to temporary symptoms rather than a diagnosis based on DSM-V criteria such as Post-Traumatic Stress Disorder. I don't think it's wrong to include post-traumatic stress as a maternal mental health outcome of study, but I would recommend further defining it in the introduction and making the distinction between this condition and an actual PTSD diagnosis.

We thank the reviewer for this thoughtful comment and agree that distinguishing between post-traumatic stress symptoms and a formal diagnosis of Post-Traumatic Stress Disorder (PTSD) is important. In our study, “post-traumatic stress” was defined using ICD-9 code, “3098” and ICD-10 codes, “F43.0”, “F43.1”, “F43.2”, “F43.8”, “F43.9”), which include a spectrum of trauma- and stressor-related disorders, including acute stress reactions, PTSD, and adjustment disorders. The DSM-V criteria for PTSD requires that symptoms persist for one month, so restricting to these alone would miss people who had potentially severe, traumatic responses to childbirth that occurred in the first month postpartum.

To improve clarity, we have revised the “Materials and Methods” section to explicitly define “post-traumatic stress” as a broad category capturing both PTSD diagnoses and related trauma- and stressor-related conditions, rather than exclusively DSM-V-defined PTSD.

> Lines 132-137: The conditions were depression, anxiety, post-traumatic stress (acute stress reaction, post-traumatic stress disorder, adjustment disorder, other reactions to severe stress), and other serious mental illnesses (bipolar spectrum disorders, schizophrenia spectrum disorders, psychotic disorders, delusional disorders and personality disorders), identified using ICD codes from inpatient and outpatient encounters (Supplemental Table 2)[19,20]
>
> Lines 140-142: We evaluated the broad category of post-traumatic stress (rather that PTSD specifically) to comprehensively assess all potentially severe, traumatic responses to childbirth regardless of time of onset (e.g. PTSD requires symptoms present for over one month).

2. For lines 72-82, if you are trying to make the argument that perhaps obstetricians are shying away from OVD because of concerns over creating trauma, I think the comparison between prevalence of OVD in the US versus the UK and other countries can be left out. I am not sure what it adds to this argument.

Thank you for this insightful comment. We agree that the comparison to operative vaginal delivery rates in other countries does not directly strengthen our argument regarding concerns about obstetric trauma. We have therefore removed this comparison and revised the paragraph to maintain focus.

Lines 65-81: Operative vaginal deliveries, including vacuum and forceps assisted deliveries, are an important component of labor management as they can expedite delivery for maternal or fetal indications while avoiding a second-stage cesarean delivery and its associated morbidities[9]. OVDs account for 3.4% (2.4 – 2.6% vacuum- assisted deliveries, 0.4- 0.5% forceps-assisted deliveries) of all deliveries in the United States (2000-2018)[9–11]. This discrepancy has been attributed to decreased contemporary provider training and comfort, and higher threshold for operative vaginal delivery intervention where cesarean delivery may be favored[10,11,13]. Concern exists that the experience of an OVD attempt can be traumatic for birthing individuals, particularly when the goal is to expedite delivery in an emergent situation and the OVD attempt is unsuccessful. Prior studies have been limited and yielded contradictory findings in regards to mental health outcomes with OVD[2,6,14]. Robust, contemporary data evaluating the mental health consequences of all modes of delivery (including successful and unsuccessful OVD attempts) are critically needed. To address these knowledge gaps, we evaluated the association of mode of delivery with incident postpartum psychiatric diagnoses, with the goal of delineating how delivery interventions may influence postpartum psychiatric outcomes. We hypothesized that cesarean deliveries with and without OVD attempts, but not successful OVDs, would have increased rates of postpartum psychiatric conditions compared with spontaneous vaginal birth.

3. In line 102, why were "unusual or rare forceps deliveries" excluded? It would be nice to see this similarly addressed to how the other inclusion/exclusion criteria are explained.

Thank you for this helpful suggestion. We would like to clarify that cases of unusual or rare forceps deliveries (e.g. rotational forceps) were excluded because they represent atypical clinical scenarios that are not comparable or generalizable to standard contemporary operative vaginal deliveries. This also raises concern for information bias, as it questions the validity of the codes. We have added language to the "Materials and Methods" section to clarify this rationale. Approximately 1000 deliveries were excluded for rare or unusual forceps (32 with forceps used for aftercoming head, 555 with mid or high forceps, 294 with rotational forceps, and 145 with combined vacuum and forceps).

Lines 99-103: We also excluded unusual or rare forceps deliveries, such as forceps used for aftercoming head, mid or high forceps, rotational forceps, and combined vacuum and forceps deliveries, as these represent atypical clinical scenarios that are not comparable or generalizable to standard, contemporary operative vaginal deliveries. Their inclusion would also raise concern for information bias, as it questions the validity of the codes.

4. I am a little confused by lines 116-119. Were only the cesareans with an accompanying code for placenta prevue, vasa previa, and fetal malpresentation included in the planned cesarean group? I think this could be reworded to read something like "the planned cesarean group was comprised of patients who had both a code for cesarean and a code for a diagnosis contraindicating vaginal delivery, including placenta prevue, vasa previa, and fetal malpresentation". You could also consider including patients with a prior classical.

Thank you for this helpful suggestion. We agree that the description of how planned cesarean deliveries were defined requires clarification. Planned cesareans were not limited to cases with coding for placenta previa, vasa previa, or fetal malpresentation. We additionally included all deliveries with a cesarean code, and no labor code and no operative vaginal delivery attempt coding in the planned cesarean cohort. We have clarified this point in the "Materials and Methods" section. While we agree a prior classical uterine incision should be included in the planned cesarean group, there is no ICD or CPT code that distinguishes a "classical" cesarean section from other incision types so we were unable to assess this.

We would also like to note that, while we attempted to classify mode of delivery as accurately as possible, the distinction between planned and unplanned cesarean is limited by administrative codes. This has been added to the limitations.

> Lines 124-130: Deliveries were classified as planned cesareans if they had both a cesarean delivery code and a code indicating a contraindication to vaginal delivery (e.g., placenta previa, vasa previa, or fetal malpresentation). Cases in which these contraindication codes were present without a corresponding cesarean code, or in conjunction with a code indicating an operative vaginal delivery (OVD) attempt, were excluded. Additionally, deliveries with a cesarean code but no labor-related codes or OVD attempt were also classified as planned cesareans (Appendix 2).

> Lines 283-292: Our study is additionally limited by the use of billing codes, which are subject to misclassification, under ascertainment, and temporal changes in screening, referral and diagnostic practices over the study period. Specifically, assessment of unplanned, intrapartum cesarean births – particularly those in the second stage – is challenging based on lack of well-defined corresponding codes to distinguish planned versus unplanned cesareans. We addressed by developing an algorithm to exclude conditions for which cesareans are typically planned, as we have done previously[18]. Despite these attempts to classify mode of delivery as accurately as possible, the distinction between planned and unplanned cesarean remains limited by available data.

5. I am not sure if I would include BPD, schizophrenia, and personality disorders as part of your composite outcome. These disorders, to my understanding, are unlikely to emerge as a direct result of experiencing traumatic birth and perhaps more likely to be unmasked by the stressors accompanying new parenthood (like lack of sleep). They are also quite uncommon.

We appreciate the reviewer's comment regarding the inclusion of borderline personality disorder (BPD), schizophrenia, and other personality disorders in our composite psychiatric outcome. We elected to include them in the composite outcome for completeness and consistency with prior literature examining severe postpartum psychiatric morbidity, acknowledging that they are rare. We would also like to note that there is increasing recognition that bipolar disorder is a prevalent, under-recognized psychiatric comorbidity that frequently exacerbates or presents in the pregnant or postpartum period[1,2]. For example, a study by our co-author, Dr. Wisner, previously demonstrated that 22.6% of postpartum women with positive

depressive screens (EPDS scores of 10 or higher) had comorbid bipolar disorder on further evaluation.[3]

Reviewer comment references:

[1] Pope CJ, Sharma V, Mazmanian D. Bipolar Disorder in the Postpartum Period: Management Strategies and Future Directions. *Womens Health (Lond Engl)*. 2014;10(4):359-371. doi:10.2217/WHE.14.33

[2] Prevalence of Bipolar Disorder in Perinatal Women: A Systematic Review and Meta-Analysis - PMC. Accessed March 25, 2026. https://pmc.ncbi.nlm.nih.gov/articles/PMC10849873/

[3] Wisner KL, Sit DK, McShea MC, Rizzo DM, Zoretich RA, Hughes CL, Eng HF, Luther JF, Wisniewski SR, Constantino ML, Confer AL, Moses-Kolko EL, Famy CS, Hanusa BH. Onset timing, thoughts of self-harm, and diagnoses in postpartum women with screen-positive depression findings. JAMA Psychiatry 70(5): 490-8, 2013. PMID: 23487258. Doi: 10.1001/jamapsychiatry.2013.87

6. Did you consider adding pregnancy induced hypertensive disorders to your covariates, or postpartum hemorrhage, both of which can be independent causes of trauma? They might be worth including as potential confounders.

Thank you for this thoughtful suggestion. We did not include these variables in our primary model because our analytic approach aimed to adjust for baseline and antepartum characteristics rather than intrapartum or peripartum complications that may mediate the causal pathway between delivery experience and onset of subsequent psychiatric conditions. We agree that assessing these distinct factors would be an important next step in research, especially given our results around cesarean delivery. In response to this comment, we have expanded our Discussion on as shown below.

> Lines 255-265: While our study highlights important associations, further research is needed to clarify the underlying mechanisms, identify potential protective factors and modifiable risk factors. Postpartum psychiatric conditions are likely influenced by a range of downstream factors independent of mode of delivery that warrant further investigation. These include neonatal outcomes, maternal complications, and psychosocial determinants. Improved characterization of these factors may help elucidate additional mechanisms underlying postpartum psychiatric conditions and identify additional targets for intervention. Future studies that incorporate patient perspectives and examine moderating variables may deepen our understanding of why cesareans are associated with higher rates of postpartum psychiatric conditions. Gaining clearer insight into these mechanisms would support the development of targeted early clinical interventions to better serve this at-risk population.

7. For line 193, my understanding is that you included cesareans formed for malpresentation- if this is the case, I would recommend removing "vertex-presenting" from this line. If I somehow

misunderstood and you did exclude patients who had a planned CS for malpresentation, this bears explaining.

Thank you for this comment. Cesarean deliveries performed for malpresentation were included in our planned cesarean cohort. We have revised the text to remove "vertex-presenting" for clarity.

> Lines 207-210: In this study of nearly one million singleton, pregnancies across the United States, new postpartum psychiatric conditions were more frequently diagnosed in those undergoing cesarean births regardless of whether they were planned or unplanned, and with or without an OVD attempt.

8. For lines 243-244, is there a reason you suspect that social support (presumably decreased support) underlies the higher rate of mental health diagnoses seen in your study? If not, I would remove the hyphenated clause. If so, I would flesh this out more. It's been well-demonstrated that limited social support is a risk factor for adverse mental health outcomes, but I am not sure it's plausible as a moderating variable that would contribute to the higher rates of mental health disorder seen in patients undergoing cesarean.

Thank you for this insightful comment. We agree our mention of social support as a potential contributor was not sufficiently supported. We have removed the hyphenated clause to maintain the focus of paragraph.

> Lines 261-265: Future studies that incorporate patient perspectives and examine moderating variables may deepen our understanding of why cesareans are associated with higher rates of postpartum psychiatric conditions.

9. I would recommend in some way addressing the way that you handled TOLAC (which could simply be to mention that you were limited by the information included in the ICD codes and were likely not able to accurately separate TOLAC cases, which might have their own inherent risk factors for postpartum mental health disorders) and the way that you handled the COVID-19 pandemic (which it appears you did by controlling for the delivery year) in your discussion somewhere.

While there is no code available that is specific to TOLAC, we have added the code for previous cesarean (ICD9 654.2 and ICD10 O34.21) to Table 1. This code was present in 7.2% of births in our cohort overall.

In regards to the comment about the COVID-19 pandemic, we have specified that adjusting for delivery year is partially intended to account for mental health effects from the COVID-19 pandemic in the Methods as shown below:

> Lines 145-150: We selected covariates including age, region of residence, and delivery year, as well as conditions that increase the risk of cesarean delivery, including class III

obesity, chronic hypertension, and diabetes (type 1 or 2)[23]. We adjusted for delivery year to account for temporal changes, including potential impacts of the COVID-19 pandemic. For substance use, we included codes for alcohol and drug use during pregnancy (tobacco, hallucinogens, cocaine, amphetamines, inhalants, or other unspecified drug use) (Appendix 1).

Table 1. Clinical characteristics and birth outcomes of individuals with term singleton, live births who underwent a trial of labor in the United States between 2008 and 2022 (N= 934,524).

	Spontaneous vaginal delivery	Successful operative vaginal delivery (OVD)	Planned Cesarean	Unplanned cesarean delivery, no OVD attempt	Cesarean after failed OVD	p-value[§]
	N = 618,594	N= 42,860	N= 130,571	N= 135,808	N= 6,691	
Sociodemographic Characteristics						
Age, years						<0.001
< 25	36,793 (6%)	2,906 (7%)	3,826 (3%)	7,085 (5%)	314 (5%)	
25-29	186,915 (30%)	14,435 (34%)	28,203 (22%)	37,784 (28%)	1,907 (29%)	
30-34	259,048 (42%)	17,098 (40%)	55,817 (43%)	55,280 (41%)	2,761 (41%)	
35-29	115,698 (19%)	7,041 (16%)	34,256 (26%)	28,476 (21%)	1,378 (21%)	
40+	20,140 (3%)	1,380 (3%)	8,469 (7%)	7,183 (5%)	331 (5%)	
Region of the U.S.						<0.001
North Central	153,451 (25%)	11,393 (27%)	28,081 (22%)	30,005 (22%)	2,122 (32%)	
Northeast	108,225 (17%)	6,914 (16%)	20,900 (16%)	24,330 (18%)	907 (14%)	
South	244,383 (40%)	16,664 (39%)	62,233 (48%)	60,600 (45%)	2,439 (36%)	
West	105,324 (17%)	7,391 (17%)	17,899 (14%)	19,184 (14%)	1079 (16%)	
Delivery Year						<0.001
2008-2012	278,545 (45%)	21,050 (49%)	47,210 (36%)	62,043 (46%)	3,916 (59%)	
2013-2017	207,979 (34%)	13,518 (32%)	46,375 (36%)	45,867 (34%)	2,186 (33%)	
2018-2022	132,070 (21%)	8,292 (19%)	36,989 (28%)	27,898 (21%)	589 (8.8%)	
Clinical Characteristics and Birth Outcomes						
BMI ≥ 40 kg/m^2	8,028 (1%)	435 (1%)	4,169 (3%)	4,513 (3%)	164 (3%)	<0.001
Substance Use During Pregnancy[*]	3,886 (1%)	292 (1%)	924 (1%)	1,182 (1%)	43 (1%)	<0.001
Chronic Hypertension	20,967 (3%)	1,508 (4%)	7,542 (6%)	8,575 (6%)	437 (7%)	<0.001
Diabetes (type 1 or 2)	16,408 (3%)	1,206 (3%)	5,688 (4%)	6,385 (5%)	371 (6%)	<0.001
Gestational hypertension or preeclampsia without severe features	58,055 (9%)	4,611 (11%)	15,251 (12%)	20,694 (15%)	848 (13%)	<0.001
Preeclampsia with severe features	7,691 (1%)	655 (2%)	3,332 (3%)	4,467 (3%)	161 (2%)	<0.001
Mean Gestational Age at Delivery (weeks; SD)	39.0 (0.6)	39.0 (0.5)	38.9 (0.5)	39.0 (0.6)	39.0 (0.4)	<0.001
Induction of Labor	121,752 (20%)	9,300 (22%)	3,503 (3%)	21,961 (16%)	845 (13%)	<0.001
Postpartum Hemorrhage	19,173 (3%)	1,719 (4%)	2,227 (2%)	3,194 (2%)	169 (3%)	<0.001
Intra-amniotic Infection	13,551 (2%)	2,387 (6%)	1,859 (2%)	8,471 (6%)	293 (4%)	<0.001
Previous Cesarean Delivery	8,789 (13%)	775 (1%)	36,519 (54%)	20,181 (30%)	1,422 (2%)	<0.001
Severe maternal morbidity[†]	5,039 (1%)	589 (1%)	2,293 (2%)	2,947 (2%)	154 (2%)	<0.001
Severe unexpected complications in term newborns [‡]	11,220 (2%)	1,773 (4%)	2,244 (2%)	4,829 (4%)	227 (3%)	<0.001

N (column %) unless stated otherwise

*Substance use included alcohol, tobacco, hallucinogens, cocaine, amphetamines, inhalants, or other unspecified drug use

† Severe maternal morbidity index, as defined by the Center for Disease Control, 21 indicators with associated ICD codes, including transfusion.

‡ Includes any of the following: severe birth trauma, severe hypoxia/asphyxia, severe shock and resuscitation, neonatal severe respiratory complications, neonatal severe infection, neonatal severe neurological complications, neonatal severe septicemia, severe shock and resuscitation procedures, neonatal severe respiratory procedures, neonatal severe neurological procedures

§ Categorical variables were compared using Pearson’s chi-squared test, and continuous variables were compared using the Kruskal-Wallis rank sum test

Reviewer #3: The authors have presented a very large study showing association between mode of delivery and psychiatric illness post-partum.

1. The limitation that the sample although huge consists of only women who have private insurance and sought help for the psychiatric symptoms is stated. The sample depends on accurate coding and this may decrease generalizability.

Thank you for highlighting this important limitation. We acknowledge that our dataset may limit generalizability and is dependent on accurate coding practices. We have included these limitations in the "Discussion". We note that the large dataset with broad geographic representation across the United States allowed for evaluation of a relatively rare exposure (operative vaginal delivery) and assessment of multiple distinct psychiatric outcomes beyond depression. This enabled us to expand on prior literature investigating mode of delivery and psychiatric outcomes.

> Lines 281-283: A limitation of our study is the population consisted only of individuals who had commercial insurance and received healthcare for psychiatric symptoms. This could underestimate the potential effects of traumatic delivery mode on postpartum mental health.

STATISTICAL EDITOR COMMENTS:

1. Lines 102-104. Is the exclusion of these types of operative deliveries normally done in the literature? How many were excluded due to this reason? It may be worth adding sensitivity analyses with these participants included, they may be more likely to experience the outcome.

Thank you for your question. Approximately 1000 deliveries were excluded for rare or unusual forceps (32 with forceps used for aftercoming head, 555 with mid or high forceps, 294 with rotational forceps, and 145 with combined vacuum and forceps). With this small of a sample size, a sensitivity analysis including these deliveries is unlikely to impact the outcomes.

2. Lines 117-118. This doesn't appear to be an exhaustive list of the criteria for planned cesarean deliveries. If there was a cesarean delivery without a trial of labor and no placenta previa, etc. were they excluded? The exclusion criteria may need to be updated.

Thank you for this comment and for bringing the text to our attention for correction. We now clarify here and in the revised manuscript that planned cesareans were not limited to cases with coding for placenta previa, vasa previa, or fetal malpresentation. We additionally included all deliveries with a cesarean code, and no labor code and no operative vaginal delivery attempt coding in the planned cesarean cohort. We have clarified this point in the "Materials and Methods" section. Please see response above to Reviewer 2, Comment 4 for additional details regarding changes made.

> Lines 124-130: "Deliveries were classified as planned cesareans if they had both a cesarean delivery code and a code indicating a contraindication to vaginal delivery (e.g.,

placenta previa, vasa previa, or fetal malpresentation). Cases in which these contraindication codes were present without a corresponding cesarean code, or in conjunction with a code indicating an operative vaginal delivery (OVD) attempt, were excluded. Additionally, deliveries with a cesarean code but no labor-related codes or OVD attempt were also classified as planned cesareans (Appendix 2).

3. Lines 165-170 and Table 1. Statistical tests should be added to the analytical plan to describe significant differences in characteristics between exposure groups.

Thank you for this helpful suggestion. We have revised the analytical plan and Table 1 to include the statistical tests used for group comparisons. Specifically, categorical variables were analyzed using Pearson's chi-squared test, and continuous variables using the Kruskal–Wallis rank-sum test. This has been added to the Methods. The revised Table 1 is pasted above in Reviewer 2, Comment 9.

Lines 151-153: We first compared the characteristics of individuals by mode of delivery using Pearson's chi-squared test for categorical variables and Kruskal-Wallis rank sum test for continuous variables.

4. Lines 171-173. Data for the entire cohort is not presented in Figure 2.

Thank you for raising this point. Figure 2 is intended to demonstrate the prevalence of each mental health condition within each mode of delivery category, so the percentages should not total 100%. Rather, percentages in Figure 2 should match the frequencies in Table 2. The Figure 2 caption has been edited for clarification: "Figure 2: Prevalence of postpartum psychiatric conditions within each mode of delivery category".

5. Lines 175-177. Adjusted estimates are presented, the text should state that the results were obtained after adjusting for covariates.

We have added this to the text and note that Appendix 4 includes the crude, unadjusted risk ratios for Table 2.

Lines 189-193: The incidence of any psychiatric diagnosis in the first 6 months after delivery was increased in individuals who underwent a planned cesarean (11.4%; aRR: 1.19 (95% CI: 1.17-1.21)), unplanned cesarean without OVD attempt (10.8%; aRR: 1.16 (95% CI: 1.14-1.18)), and unplanned cesarean delivery with failed OVD attempt (10.9%; aRR: 1.26 (95% CI: 1.18-1.35) compared with people who had an SVD (9.2%) (Table 2, Appendix 4).

6. Line 177. The aRR should be 1.26.

Thank you – this is now corrected.

Lines 189-194: In analyses both with and without adjustment for confounders, compared with people who had an SVD (9.2%), the incidence of any psychiatric diagnosis in the

first 6 months after delivery was increased in individuals who underwent a planned cesarean 11.4%, (aRR 1.19 (95% CI, 1.17 – 1.21)), unplanned cesarean without OVD attempt 10.8%, (aRR 1.16 (95% CI, 1.14 -1.18)), and unplanned cesarean delivery with failed OVD attempt 10.9%, (aRR 1.26 (95% CI, 1.18 -1.35)), (Table 2, Appendix 4).

7. Table 1. Include the covariates in the footnote.

The covariates included in the models have been added to the footnote.

Table 2. Adjusted risk ratios for new-onset postpartum psychiatric conditions by mode of delivery among term, singleton, livebirths undergoing a trial of labor in a commercial claims database (N=934,524) [§].

	SVD (Reference)	Successful OVD*		Planned Cesarean		Unplanned cesarean with no OVD attempt		Cesarean after failed OVD	
	N = 618,594	N = 42,860	aRR*,† (95% CI)	N= 130,571	aRR † (95% CI)	N = 135,808	aRR † (95% CI)	N = 6,691	aRR † (95% CI)
Any psychiatric condition	57,191 (9.2%)	3,936 (9.2%)	1.00 (0.97, 1.03)	14,957 (11.4%)	1.19 (1.17, 1.21)	14,747 (10.8%)	1.16 (1.14, 1.18)	731 (10.9%)	1.26 (1.18, 1.35)
Depression	38,550 (6.2%)	2,693 (6.3%)	1.00 (0.96, 1.04)	9,787 (7.5%)	1.19 (1.16, 1.22)	9,983 (7.4%)	1.17 (1.14, 1.19)	509 (7.6%)	1.23 (1.13, 1.34)
Anxiety	20,958 (3.4%)	1,372 (3.2%)	0.97 (0.92, 1.03)	5,691 (4.4%)	1.18 (1.14, 1.21)	5,424 (4.0%)	1.18 (1.14, 1.21)	249 (3.7%)	1.30 (1.15, 1.47)
Post - Traumatic Stress	4,559 (0.7%)	324 (0.8%)	1.09 (0.97, 1.22)	1,351 (1.0%)	1.12 (1.06, 1.19)	1,111 (0.8%)	1.10 (1.03, 1.18)	43 (0.6%)	1.40 (1.04, 1.89)
Serious Mental Illness ‡	1,239 (0.2%)	82 (0.2%)	0.93 (0.74, 1.16)	311 (0.2%)	1.25 (1.10, 1.42)	333 (0.2%)	1.19 (1.06, 1.35)	16 (0.2%)	1.14 (0.69, 1.86)

* Abbreviations: OVD = Operative Vaginal Delivery, aRR = Adjusted Risk Ratio. Numbers shown as N (column percent).
† Poisson regression model with robust standard errors
‡ Serious mental illnesses included bipolar spectrum disorders, schizophrenia spectrum disorders, psychotic disorders, delusional disorders and personality disorders
§ Models adjusted for maternal age, region, delivery year, class III obesity, substance use, gestational age at delivery, chronic hypertension, pre-pregnancy diabetes (type I or type II).

8. Lines 187-189. This data is not in the table. If elsewhere, please state the location.

The results for the sensitivity analysis using GEE models now presented in Appendix 6.

Appendix 6: Sensitivity analysis of post-traumatic stress during the ICD-10 years only (2016-2022)

	Successful OVD[*]		Planned Cesarean Delivery		Unplanned cesarean delivery with no OVD attempt		Cesarean delivery after failed OVD	
	RR[*†] (95% CI)	aRR[*†] (95% CI)	RR (95% CI)	aRR (95% CI)	aRR (95% CI)	aRR (95% CI)	RR (95% CI)	aRR (95% CI)
Post Traumatic Stress	1.12 (0.99, 1.26)	1.10 (0.97, 1.24)	1.08 (1.01, 1.15)	1.07 (1.00, 1.14)	1.14 (1.07, 1.23)	1.11 (1.03, 1.19)	1.53 (1.05, 2.22)	1.45 (1.00, 2.10)

[*]Abbreviations: OVD = Operative Vaginal Delivery, RR = Risk Ratio, aRR = Adjusted Risk Ratio.

[†] Relative risk and adjusted relative risk compared to referent group of spontaneous vaginal delivery
