# Supplementary material for: Association between delivery mode and postpartum psychiatric conditions: A national cohort study
Source: Obstet Gynecol. Author manuscript; Available in PMC 2026 Aug 6. (PMC13439854; doi:10.1097/AOG.0000000000006321)
Supplement: Supplementary file 1 [file NIHMS2190408-supplement-Appendices_1-6.docx]

Appendix 1. ICD diagnosis codes used to classify independent and dependent variables. 

| **Diagnosis** | **ICD-9** | **ICD-10** |
| --- | --- | --- |
| Depressive Disorders | “2962” “2963” “2968” “3004” “3091” “311” | “F320” “F321” “F323” “F324” “F325” “F328” “F329” “F330” “F331” “F332” “F333” “F334” “F338” “F339” “F341” “F530” |
| Anxiety Disorders | “2938” “3000” “3002” “3003” | “F410” “F411” “F412” “F413” “F418” “F419” “F400” “F401” “F402” “F408” “F409” |
| Post traumatic stress symptoms | “3098” | “F430” “F431” “F432” “F438” “F439” |
| Other serious mental health condition | “2960” “2961” “2964” “2965” “2966” “2967” “2968” “2969” “2950” “2951” “2952” “2953” “2954” “2955” “2956”  “2957” “2958” “2959” | “F201” “F202” “F203” “F205” “F208” “F208” “F209” “F22” “F23” “F24” “F250” “F251” “F258” “F259” “F28” “F29” “F310” “F311” “F312” “F313” “F314” “F315” “F316” “F317” “F318” “F319” “F30.1” “F302” “F303” “F304” “F308” “F309” “F600” “F601” “F602” “F603” “F604” “F605” “F606” “F607” “F6081” “F6089” “F609” “F531” |
| Vacuum extraction | “7271” “7279” | “O814” “10D07Z6” |
| Forceps extraction | “720” “721” | “O810” “O813” “10D07Z3” |
| Other instrumental delivery | “6607” “6695” “728” “729” | “O665” |

Appendix 2. Antidepressant medications used to define depression

| **Tricyclic Antidepressants** | **Selective Serotonin Reuptake    Inhibitors** | **Neurosteroid Antidepressant** |
| --- | --- | --- |
| Amitriptyline     Amoxapine     Desipramine     Doxepin     Imipramine     Nortriptyline     Protriptyline     Trimipramine     Clomipramine | Citalopram     Escitalopram     Fluoxetine     Fluvoxamine     Paroxetine     Sertraline     Vilazodone | Zuranolone     Brexanolone |

Appendix 3. ICD diagnosis codes used to identify comorbidities 

| **Variable** | **ICD-9** | **ICD-10** |
| --- | --- | --- |
| Class 3 obesity, BMI ≥ 40 kg/m^2^ | "27801" | "Z684" "E6601" "E662" |
| Diabetes mellitus types 1 and 2 | "250" "6480" | "E08" "E09" "E10" "E11" "E12" "E13" "O240" "O241" "O243" "O248" "O249" "Z794" |
| Chronic hypertension | “401” “6420” “6427” | “O100” “O11” “I10” |
| Gestational hypertension or mild preeclampsia | "6423" "6424" | "O13" "O140" "O149" |
| Severe preeclampsia | "6425" "6427" | "O11" "O141" "O142" |
| Substance use^*^ | “64830” “64832” “64833” “291” “292” “303” “3041” “3042” “3043” “3044”  “3045” “3046” “3048” “3049” “3050” “3051” “3052” “3053” “3056” “3057” “3059” | “O9931” “O9932” “F10” “F12” “F13” “F14” “F15” “F16” “F17” “F18” “F19” |
| Postpartum hemorrhage | “6660” “6661” “6662” | “O720” “O721” “O722” |
| Intraamniotic infection | "6584" | "O411” |
| Labor complication^†^ | “653” “6590” “6591” “6592” “6593” “6597” “660” “661” “662” “663” “664” “665” “6695” “6696” “6697” | “O33” “O61” “O62” “O63” “O64” “O65” “O66” “O69” “O70” “O71” “O752” “O753” “O7581” “O7582” “O76” “O77” |
| Induction of labor^†^ | “7301” “731” “734” | “3E033VJ” “0U7C7ZZ” “0U7C7DZ” “3E0P7GC” |
| Multifetal gestation | "651" "V272" "V273" "V274" "V275" "V276" "V277" | "O30" "O31" "O632" "Z372" "Z373" "Z374" "Z375" "Z376" "Z377" |
| Fetal malpresentation | “6522” “6523” “6524” “6520” “6527” “6528” “6529” “6696” | “O32” “O642” “O643” “O644” “O645” “O648” “O649” “0830” “0831” |
| Vasa previa | “6635” | “0694” |
| Placenta previa | “6410” “6411” | “O4400” “O4403” “O4410” “O4413” “O4420” “O4423” “O4440” “O4443” “O4450” “O4453” “O4430” “O4433” |
| Mid or high forceps | “772x” “723x” | “O811” “10D07Z4” “10D07Z5” |
| Rotational forceps | “724” | “O812” |
| Breech extraction or Forceps for aftercoming head | “725x” “726” | “O830” “O831” |

^*^ Substance use included alcohol, tobacco, hallucinogens, cocaine, amphetamines, inhalants, or other nonspecified drug use

^† ^The presence of any ICD-9-CM, ICD-10-CM, or CPT code within these two categories, along with a cesarean delivery procedure code, was used to identify unplanned or intrapartum cesarean deliveries.

Appendix 4. Risk ratios for new-onset postpartum psychiatric conditions by mode of delivery among term, singleton, livebirths undergoing a trial of labor in a commercial claims database (N=934,524)

|  | **Successful OVD** | **Planned Cesarean Delivery** | **Unplanned cesarean with no OVD attempt** | **Cesarean after failed OVD**^*^ |
| --- | --- | --- | --- | --- |
|  | RR ^†^ (95% CI) | RR (95% CI) | RR (95% CI) | RR (95% CI) |
| **Any Mental Health Diagnosis** | 0.99 (0.96, 1.02) | 1.24 (1.22, 1.26) | 1.17 (1.15, 1.19) | 1.18 (1.10, 1.27) |
| **Depression** | 1.01 (0.97, 1.05) | 1.20 (1.18, 1.23) | 1.18 (1.15, 1.20) | 1.22 (1.12, 1.33) |
| **Anxiety** | 0.94 (0.90, 1.00) | 1.29 (1.25, 1.32) | 1.18 (1.14, 1.21) | 1.10 (0.97, 1.24) |
| **Post Traumatic Stress** | 1.03 (0.92, 1.15) | 1.40 (1.32, 1.49) | 1.11 (1.04, 1.18) | 0.87 (0.65, 1.18) |
| **Serious Mental Illness** ^‡^ | 0.96 (0.76, 1.19) | 1.19 (1.05, 1.35) | 1.22 (1.08, 1.38) | 1.19 (0.73, 1.95) |

^*^Abbreviations: RR = risk ratio, OVD = Operative Vaginal Delivery

^†^ Risk ratio of postpartum mental condition in comparison to spontaneous vaginal delivery (referent group) 

^‡ ^ Serious mental illnesses included bipolar spectrum disorders, schizophrenia spectrum disorders, psychotic disorders, delusional disorders and personality disorders

Appendix 5: Sensitivity analysis of postpartum mental conditions by mode of delivery, using Generalized Estimating Equations (GEE) to account for clustering of multiple births per person.

|  | Successful OVD^*^ |  | Planned Cesarean Delivery |  | Unplanned cesarean delivery with no OVD attempt |  | Cesarean delivery after failed OVD |  |
| --- | --- | --- | --- | --- | --- | --- | --- | --- |
|  | RR^*†^ (95% CI) | aRR^*†^ (95% CI) | RR (95% CI) | aRR (95% CI) | aRR (95% CI) | aRR (95% CI) | RR (95% CI) | aRR (95% CI) |
| Any Mental Condition | 0.99 (0.96, 1.02) | 1.00 (0.97, 1.03) | 1.24 (1.22, 1.26) | 1.18 (1.16, 1.20) | 1.17 (1.15, 1.19) | 1.16 (1.14, 1.18) | 1.18 (1.10, 1.26) | 1.26 (1.17, 1.35) |
| Depression | 1.00 (0.96, 1.04) | 1.00 (0.96, 1.03) | 1.20 (1.18, 1.23) | 1.19 (1.16, 1.21) | 1.17 (1.15, 1.20) | 1.16 (1.14, 1.19) | 1.22 (1.12, 1.32) | 1.22 (1.12, 1.32) |
| Anxiety | 0.94 (0.89, 0.99) | 0.97 (0.92, 1.02) | 1.28 (1.25, 1.32) | 1.17 (1.14, 1.21) | 1.17 (1.14, 1.21) | 1.17 (1.14, 1.21) | 1.10 (0.97, 1.24) | 1.30 (1.15, 1.47) |
| Post Traumatic Stress | 1.02 (0.92, 1.15) | 1.09 (0.97, 1.22) | 1.40 (1.32, 1.49) | 1.12 (1.06, 1.19) | 1.11 (1.04, 1.18) | 1.10 (1.03, 1.18) | 0.87 (0.65, 1.18) | 1.40 (1.04, 1.89) |
| Serious Mental Illness ^‡^ | 0.96 (0.76, 1.19) | 0.93 (0.74, 1.16) | 1.19 (1.05, 1.34) | 1.25 (1.10, 1.42) | 1.22 (1.08, 1.38) | 1.19 (1.05, 1.35) | 1.19 (0.73, 1.95) | 1.14 (0.70, 1.86) |

^*^Abbreviations: OVD = Operative Vaginal Delivery, RR = Risk Ratio, aRR = Adjusted Risk Ratio.  

^†^  Risk ratio and adjusted risk ratio compared to referent group of spontaneous vaginal delivery

^‡^ Serious mental illnesses included bipolar spectrum disorders, schizophrenia spectrum disorders, psychotic disorders, delusional disorders and personality disorders

Appendix 6: Sensitivity analysis of post-traumatic stress during the ICD-10 years only (2016-2022)

|  | Successful OVD^*^ |  | Planned Cesarean Delivery |  | Unplanned cesarean delivery with no OVD attempt |  | Cesarean delivery after failed OVD |  |
| --- | --- | --- | --- | --- | --- | --- | --- | --- |
|  | RR^*†^ (95% CI) | aRR^*†^ (95% CI) | RR (95% CI) | aRR (95% CI) | aRR (95% CI) | aRR (95% CI) | RR (95% CI) | aRR (95% CI) |
| Post Traumatic Stress | 1.12 (0.99, 1.26) | 1.10 (0.97, 1.24) | 1.08 (1.01, 1.15) | 1.07 (1.00, 1.14) | 1.14 (1.07, 1.23) | 1.11 (1.03, 1.19) | 1.53 (1.05, 2.22) | 1.45 (1.00, 2.10) |

^*^Abbreviations: OVD = Operative Vaginal Delivery, RR = Risk Ratio, aRR = Adjusted Risk Ratio.  

^†^  Risk ratio and adjusted risk ratio compared to referent group of spontaneous vaginal delivery
